# Genetic Interactions of Phase II Xenobiotic-Metabolizing Enzymes *GSTO1* and *GCLC* in Relation to Alcohol Abuse and Psoriasis Risk

**DOI:** 10.3390/jox15020060

**Published:** 2025-04-20

**Authors:** Roman Saranyuk, Olga Bushueva, Ekaterina Efanova, Maria Solodilova, Mikhail Churnosov, Alexey Polonikov

**Affiliations:** 1 Center for Medical Examinations and Prevention, 2 Leninsky Komsomol Avenue, 305026 Kursk, Russia; roman.saranuk@gmail.com; 2 Laboratory of Genomic Research, Research Institute for Genetic and Molecular Epidemiology, Kursk State Medical University, 18 Yamskaya Street, 305041 Kursk, Russia; olga.bushueva@inbox.ru (O.B.); pogozhevna25@mail.ru (E.E.); 3 Department of Biology, Medical Genetics and Ecology, Kursk State Medical University, 3 Karl Marx Street, 305041 Kursk, Russia; solodilovama@kursksmu.net; 4 Medvenka Central District Hospital, 68 Sovetskaya Street, 307030 Medvenka, Russia; 5 Department of Medical Biological Disciplines, Belgorod State University, 85 Pobedy Street, 308015 Belgorod, Russia; churnosov@bsuedu.ru; 6 Laboratory of Statistical Genetics and Bioinformatics, Research Institute for Genetic and Molecular Epidemiology, Kursk State Medical University, 18 Yamskaya Street, 305041 Kursk, Russia

**Keywords:** psoriasis, genetic susceptibility, oxidative stress, glutathione metabolism, glutathione S-transferase omega 1, *GSTO1*, single nucleotide polymorphism, alcohol abuse, gene–gene interactions, gene–environment interactions

## Abstract

The present pilot study aimed to investigate whether common single nucleotide polymorphisms (SNPs) in the gene encoding glutathione S-transferase omega 1 (*GSTO1*), both individually and in combination with variants of the catalytic subunit of the glutamate cysteine ligase (*GCLC*) gene and environmental risk factors, are associated with the risk of psoriasis. The research included a total of 944 participants, comprising 474 individuals diagnosed with psoriasis and 470 healthy control subjects. Five common SNPs in the *GSTO1* gene—specifically, rs11191736, rs34040810, rs2289964, rs11191979, and rs187304410—were genotyped in the study groups using the MassARRAY-4 system. The allele rs187304410-A (OR = 0.19, 95% CI 0.04–0.86, Pperm = 0.02) and the genotype rs187304410-G/A (OR = 0.19, 95% CI 0.04–0.85, Pperm = 0.01) were found to be associated with psoriasis in females. The model-based multifactor dimensionality reduction approach facilitated the identification of higher-order epistatic interactions between the variants of the *GSTO1* and *GCLC* genes (Pperm < 0.0001). These interactions, along with the risk factor of alcohol abuse, collectively contribute to the pathogenesis of psoriasis. This study is the first to demonstrate that polymorphisms in the *GSTO1* gene, both individually and in combination with variants of the *GCLC* gene and alcohol abuse, are associated with an increased risk of psoriasis.

## 1. Introduction

Psoriasis is a chronic dermatosis characterized by immune-mediated inflammation, resulting in the formation of thickened, scaly erythematous plaques [1]. The global prevalence of psoriasis is approximately 2%, with psoriasis vulgaris, also known as plaque-type psoriasis, representing the most common subtype and accounting for around 90% of all cases [2,3]. Psoriasis is commonly understood as a complex multifactorial disorder resulting from the interplay between genetic and environmental factors [4]. In recent decades, substantial progress has been achieved in elucidating the genetic mechanisms underlying psoriasis, primarily facilitated by genome-wide association studies that have identified more than sixty regions associated with disease susceptibility [4]. These investigations have highlighted the pathogenic significance of genes involved in the activation of Th17 cells and have also uncovered rare genetic variants associated with distinct forms of psoriasis [4,5]. Numerous extrinsic factors—such as mechanical stress, air pollutants, sun exposure, medications, vaccinations, infections, and lifestyle choices—and intrinsic factors—including obesity, diabetes mellitus, dyslipidemia, hypertension, and mental stress—have been identified as triggers and exacerbating factors for psoriasis [6]. Research conducted on extensive twin cohorts indicates that genetic factors account for 60–75% of the variability in susceptibility to psoriasis, while the remaining variation is attributed to non-shared environmental influences [7]. Despite the well-established role of environmental factors in the pathogenesis of psoriasis [8], exploring the mechanisms by which these factors disrupt the body’s equilibrium and contribute to the onset and progression of the disease remains a significant challenge.

In recent years, a growing body of research has emerged that underscores the significant role of chemical exposure, particularly air pollutants, in the etiology of psoriasis. Numerous air pollutants, including polycyclic aromatic hydrocarbons, volatile organic compounds, oxides, particulate matter, ozone, heavy metals, and ultraviolet radiation, have been shown to adversely affect the skin and increase the risk of psoriasis [9,10]. A recent large prospective cohort study involving 474,055 participants demonstrated that long-term exposure to air pollution—measured in terms of nitrogen dioxide, nitrogen oxides, fine particulate matter with a diameter of less than 2.5 µm, and particulate matter with a diameter of less than 10 µm—is associated with an increased risk of psoriasis [11]. Additionally, this study found an interaction between air pollution and genetic susceptibility that jointly contributes to the risk of psoriasis [11]. Furthermore, environmental exposure to toxic metals such as barium, cesium, antimony, uranium, lead, mercury, and cadmium has been found to compromise immunity and increase inflammation, thereby predisposing individuals to the development and exacerbation of psoriasis [12,13,14,15,16].

In order to mitigate the harmful effects of chemical pollutants, the skin expresses enzymes that are involved in xenobiotic biotransformation, with glutathione S-transferases (GSTs) playing a particularly significant role [17,18]. The primary biological function of glutathione S-transferases is to facilitate the detoxification of chemicals by catalyzing their conjugation with reduced glutathione [19]. Numerous studies have demonstrated an increased expression of glutathione S-transferases in affected skin regions compared to unaffected tissues in patients with psoriasis [20,21,22]. This observation suggests, first, the critical involvement of these enzymes in the pathogenesis of the disease, and second, that the elevated levels of GSTs in the affected skin may serve a protective function by neutralizing toxic substances that accumulate in the dermal layers. Since the genetic variability of glutathione S-transferases can influence the activity and expression of these enzymes, thereby determining individual characteristics related to skin protection against chemical exposures, polymorphisms in the GST genes have become a focal point for genetic studies on psoriasis in recent years. The vast majority of studies on psoriasis have concentrated on the deletion polymorphisms of the glutathione S-transferase genes *GSTM1* and *GSTT1* [23,24,25,26,27,28,29,30,31]. These studies have demonstrated associations with susceptibility to psoriasis, its clinical features, and the efficacy of various therapeutic approaches for the condition. However, no studies to date have been conducted to investigate whether polymorphisms in the gene encoding glutathione S-transferase omega 1 (*GSTO1*) contribute to susceptibility to psoriasis. Therefore, the present pilot study aimed to determine whether common single nucleotide polymorphisms (SNPs) in the *GSTO1* gene are associated with the risk of psoriasis.

The *GSTO1* gene was selected for this study due to its high expression in skin cells (data from https://www.gtexportal.org, accessed on 10 March 2025), where it is likely to play a crucial role in the biotransformation of xenobiotics that penetrate the skin from the environment or enter the bloodstream. An additional objective was to investigate whether polymorphisms in the *GSTO1* gene, in conjunction with variants of the catalytic subunit of the glutamate cysteine ligase (*GCLC*) gene—which encodes the first enzyme in the cellular glutathione (GSH) biosynthetic pathway—contribute to a polygenic predisposition to psoriasis. Thus, the selection of genes is justified by their functionality and potential collaborative roles in the pathogenesis of psoriasis, particularly regarding their involvement in redox homeostasis and xenobiotic metabolism.

The present study found that polymorphisms in the *GSTO1* gene contribute to susceptibility to psoriasis, with differing effects observed between males and females. In particular, the allele rs187304410-A and the genotypes rs187304410-G/A and rs187304410-A/A were found to be protective against the risk of psoriasis in females. Conversely, the allele rs11191736-T was associated with an increased risk of the disease in males. Moreover, *GSTO1* polymorphisms exhibited synergistic effects on disease risk in both males and females. In addition, joint effects on disease risk were identified for polymorphisms in the *GSTO1* and *GCLC* genes. Environmental risk factors, such as alcohol abuse and cigarette smoking, also exhibited joint effects with *GSTO1* and *GCLC* polymorphisms on the risk of developing psoriasis. The present study is the first to demonstrate that polymorphisms in the *GSTO1* gene, both individually and in combination with *GCLC* polymorphisms and glutathione-depleting environmental risk factors, contribute to susceptibility to psoriasis. This study highlights the critical role of compromised glutathione detoxification mechanisms in the development of the disease.

## 2. Materials and Methods

### 2.1. Study Patients

All phases of data collection and analysis were conducted in accordance with the principles outlined in the Declaration of Helsinki. Informed written consent was obtained from human participants in compliance with protocols approved by the Ethics Review Committee of Kursk State Medical University. The study was conducted in a case–control design and involved a cohort of 944 unrelated individuals of European ancestry, primarily consisting of Russians from the Kursk region. This group included 474 patients diagnosed with psoriasis and 470 healthy controls. Patient enrollment for the study involving individuals with psoriasis occurred at Medvenka Central District Hospital in the Kursk region, the Center for Medical Examinations and Prevention in Kursk, and the Kursk Regional Multidisciplinary Clinical Hospital, covering the period from September 2018 to December 2021. The control group, comprising individuals without chronic diseases, was drawn from our previous research efforts [32,33,34]. The mean age of patients with psoriasis was 44.3 ± 13.6 years, while the mean age of individuals in the control group was 55.3 ± 6.7 years. The patients with psoriasis (252 males and 222 females) and the control group (234 males and 236 females) were matched by gender.

### 2.2. The Inclusion/Exclusion Criteria in the Study Groups and Diagnosis of Psoriasis

The inclusion criteria for the psoriasis group were as follows: (1) a diagnosis of psoriasis confirmed by certified dermatologists through the assessment of the characteristic clinical presentation of skin lesions and their specific locations [35]; (2) Slavic ethnicity; (3) age 18 years or older; and (4) consent to participate in the study. The exclusion criteria were as follows: (1) the presence of chronic infectious diseases such as HIV or hepatitis or severe chronic conditions that could predispose individuals to the onset of psoriasis; (2) non-Slavic ethnicity; (3) current pregnancy; (4) individuals undergoing biologic therapy at the time of recruitment; and (5) refusal to participate in the study. The case group comprised individuals diagnosed with various forms of psoriasis, including classic plaque psoriasis, palmoplantar psoriasis, seborrheic psoriasis, scalp psoriasis, the von Zumbusch variant of generalized pustular psoriasis, inverse psoriasis, guttate psoriasis, and erythrodermic psoriasis. Additionally, the group included patients with comorbid conditions associated with psoriasis, such as psoriatic arthritis and onychodystrophy. The Psoriasis Area and Severity Index (PASI) was utilized as a clinical tool for assessing the severity of psoriasis progression. The criteria for inclusion in the control group were established as follows: (1) absence of chronic illnesses; (2) identification as belonging to a Slavic ethnicity; (3) age of 18 years or older; and (4) provision of informed consent to participate in the study. Conversely, the exclusion criteria included (1) identification as a non-Slavic ethnicity; and (2) refusal to provide consent for participation in the study.

### 2.3. Interviewing of Patients

Participants in the study completed a validated questionnaire administered by a physician [36] to evaluate risk factors associated with psoriasis, including cigarette smoking, alcohol consumption, and decreased consumption of fresh fruits and vegetables [37]. Interest in these risk factors arises from their harmful impact on the levels of endogenous glutathione, a vital molecule employed by glutathione S-transferases in the detoxification of xenobiotics [38]. The alcohol consumption, cigarette smoking, and dietary habits of the study participants were evaluated as previously described [39,40]. Data regarding smoking status (ever/never) were collected from all participants diagnosed with psoriasis, as well as from healthy controls. Information pertaining to alcohol consumption was obtained from all patients with psoriasis and a subset of 220 individuals from the control group. Alcohol consumption patterns were evaluated based on the number of alcoholic beverages consumed weekly, as previously outlined in the literature [38,39]. In summary, participants were classified into two categories based on their reported frequency of alcohol intake: (1) individuals who consumed alcohol 1 to 2 days per month or less, and (2) those who consumed alcohol on 1 or more days per week. The latter group was identified as alcohol abusers.

### 2.4. SNP Selection

Single nucleotide polymorphisms of the *GSTO1* gene were selected for this study based on the following criteria: (1) a minor allele frequency of ≥1% in the European population (Ensembl data, www.ensembl.org, accessed on 4 February 2023); (2) the presence of an SNP with *GSTO1* expression quantitative trait loci (eQTL) and/or splicing quantitative trait loci (sQTL) in the skin, specifically in sun-exposed areas (lower leg) and non-sun-exposed areas (suprapubic), according to data from the GTEx portal (https://gtexportal.org, accessed on 16 December 2024); (3) linkage disequilibrium (r^2^ < 0.8) among the SNPs of interest (HapMap data for the European population); and (4) the feasibility of joint SNP detection in a multiplex panel using the MALDI-TOF mass spectrometry method. As a result, five common SNPs of the *GSTO1* gene were selected for genotyping: rs11191736, rs34040810, rs2289964, rs11191979 (tagSNP), and rs187304410.

### 2.5. Genetic Analysis

All genetic investigations were performed at the Research Institute for Genetic and Molecular Epidemiology, situated at Kursk State Medical University in Kursk, Russia. Venous blood samples were obtained from the cubital vein of the study participants and collected in EDTA-coated tubes. These samples were promptly frozen and stored at −20 °C until they were processed. Genomic DNA was isolated utilizing the conventional methods of phenol/chloroform extraction followed by ethanol precipitation. Genotyping of the selected polymorphisms was conducted using the MALDI-TOF mass spectrometry iPLEX platform on the MassArray-4 system (Agena Bioscience, Inc., San Diego, CA, USA). The methodologies for sample preparation and single nucleotide polymorphism (SNP) genotyping using the MassArray-4 system are thoroughly detailed in our latest publication [41]. The primer sequences utilized for genotyping can be provided upon request. In order to ensure quality control, 5% of the DNA samples were subjected to duplicate genotyping, with the researchers remaining unaware of the case–control status. The concordance rate of the control genotyping was greater than 99%.

### 2.6. Statistical and Bioinformatics Analysis

The estimation of statistical power for the association analysis was conducted using the GAS power calculator, as documented in previous research [39]. In particular, it was estimated that the overall analysis, which included 474 cases and 470 controls, would enable the detection of a genotype relative risk (GRR) ranging from 1.30 to 1.45, with a statistical power of 82% to 98%. Furthermore, in the analysis of groups stratified by sex and risk factors, a GRR of 1.40 to 1.50 could be detected with a power of 76% to 83%, utilizing a significance level of α = 0.05. Fisher’s exact test was employed to evaluate the distribution of genotype frequencies in relation to the Hardy–Weinberg equilibrium (HWE). The frequencies of alleles and genotypes within the study cohorts, along with their correlations to the risk of developing psoriasis, were analyzed using PLINK software version 1.9 [42]. Logistic regression analysis was employed to investigate the relationships between polymorphisms in the *GSTO1* gene and the risk of psoriasis. The unadjusted odds ratios (OR) and 95% confidence intervals (95% CI) were calculated to assess the associations between single nucleotide polymorphisms (SNPs) and the corresponding phenotypes. In the context of SNP–disease associations, various genetic models—including allelic, recessive, dominant, and log-additive models—were assessed. Genetic models reflect how alleles at a specific single nucleotide polymorphism interact to influence disease phenotypes. In particular, the allelic model implies a comparison of allele frequencies between cases and controls. Chi-square tests were conducted utilizing a 2 × 2 contingency table to assess both dominant and recessive genetic models. For the log-additive model, Cochran–Armitage trend tests were employed with a 2 × 3 contingency table. A model with the lowest *p*-value was selected for interpreting the SNP–disease association. The replication of associations between polymorphisms in the *GSTO1* gene and psoriasis was conducted using the Gene ATLAS database from the UK Biobank (http://geneatlas.roslin.ed.ac.uk, accessed on 10 September 2024). Additionally, haplotype analysis of the *GSTO1* gene was conducted using Haploview software, version 4.2. *p*-Values (Pperm) for associations involving alleles, genotypes, and haplotypes were calculated using adaptive permutation methods with PLINK and Haploview software. The relationships between pairwise genotype combinations (diplotypes) and the risk of developing psoriasis were evaluated using the chi-squared test using the STATISTICA 13.0 software. Gene–gene (G × G or SNP–SNP) and gene–environment (G × E) interactions were analyzed using a model-based multifactor dimensionality reduction method (mbmdr) [43]. This non-parametric, model-free bioinformatics approach enables the identification of non-additive interactions among discrete variables, such as SNPs and risk factors that influence the development of psoriasis. The mbmdr methodology facilitates the simultaneous evaluation of multiple variables, including gene polymorphisms and environmental factors, by reducing the dimensionality of the calculated parameters [43]. By detecting complex interactions between environmental and genetic factors contributing to disease risk, this method can reveal relationships that conventional parametric statistics may overlook [43]. Among the environmental risk factors contributing to psoriasis, particular attention has been directed toward those that adversely affect endogenous levels of glutathione. These factors include excessive alcohol consumption, tobacco use, and a dietary deficiency of fresh fruits and vegetables [39]. Regarding the risk of psoriasis, we evaluated second-, third-, and fourth-order G × G and G × E models that included the *GSTO1* and *GCLC* gene polymorphisms, along with the mentioned risk factors. The mbmdr software version 3.5.3 enables the comparison of high- and low-risk mbmdr models between case and control groups, thereby identifying the optimal n-th-order G × G and G × E interactions with the lowest Pperm values that contribute to the risk of psoriasis. The overall impact of each polymorphism in the studied genes, along with environmental factors, on the polygenic predisposition to psoriasis was evaluated by aggregating all mbmdr models that included a specific genetic variant or risk factor.

## 3. Results

### 3.1. Association Between GSTO1 Gene Polymorphisms and the Risk of Psoriasis

The genotype frequencies for all single nucleotide polymorphisms of *GSTO1* were found to be in HWE in both the case and control groups (*p* > 0.05). We conducted an analysis of the relationships between polymorphisms in the GSTO1 gene and the risk of developing psoriasis, examining both the overall population and subgroups stratified by sex. A summary of the associations between *GSTO1* gene polymorphisms and psoriasis risk for both the entire cohort and the sex-stratified groups is presented in Table 1. No statistically significant associations were found between SNPs of the *GSTO1* gene and the risk of developing psoriasis when comparing the overall case and control groups. The sex-stratified analysis presented in Table 1 indicates that two specific polymorphisms are significantly associated with the risk of psoriasis: rs11191736 (allelic model) in males and rs187304410 (dominant model) in females.

Table 2 presents the genotype and allele frequencies of the *GSTO1* gene, along with their associations with the risk of psoriasis, analyzed for both the entire group and stratified by sex. In the male groups, allele rs11191736-T was found only among patients with psoriasis (Pperm = 0.017). Allele rs187304410-A (OR = 0.19, 95% CI 0.04–0.86, Pperm = 0.02) and genotype rs187304410-G/A (OR = 0.19, 95% CI 0.04–0.85, Pperm = 0.01) were found to confer protection against the risk of psoriasis in females.

Subsequently, we conducted a replication analysis to investigate the associations between polymorphisms of the *GSTO1* gene and susceptibility to psoriasis in the large cohort from the UK Biobank. None of the polymorphisms we studied demonstrated a statistically significant association with the risk of developing psoriasis in this cohort.

The difficulty in replicating associations between SNPs and diseases may be partially attributed to genetic variations across different populations, as we recently demonstrated [44,45]. This observation prompted us to conduct an association analysis of psoriasis in relation to all SNPs of the *GSTO1* gene that were genotyped within the UK Biobank cohort. As a result, 40 SNPs of the *GSTO1* gene within the UK Biobank cohort were identified as being associated with an increased risk of psoriasis, with a *p*-value of ≤0.05, as presented in Supplementary Table S1.

### 3.2. The Combined Impact of GSTO1 Gene Polymorphisms on Psoriasis Risk

The combined influence of polymorphisms in the *GSTO1* gene on the risk of developing psoriasis was assessed using haplotype and diplotype analyses. Table 3 presents the *GSTO1* haplotypes and their correlation with the risk of psoriasis, analyzed for both the overall population and stratified by sex. This study identified four prevalent haplotypes of *GSTO1*, designated as H1–H4, which exhibited a frequency exceeding 2% within the study populations. As shown in Table 3, none of the *GSTO1* haplotypes were found to be associated with the risk of psoriasis (Pperm > 0.05). SNP rs11191736 exhibited a positive linkage disequilibrium (*p* < 0.001) with the polymorphisms rs34040810 (D’ = 0.497), rs2289964 (D’ = 0.441), and rs187304410 (D’ = 0.426). Polymorphism rs34040810 was associated with rs2289964 (D’ = 0.992) and rs187304410 (D’ = 0.605). Additionally, rs187304410 showed a correlation with rs2289964 (D’ = 0.358).

Supplementary Table S2 presents the genotype combinations (diplotypes) of *GSTO1* and their associations with the risk of psoriasis. Two diplotypes, rs11191736C/C × rs34040810C/C (OR = 0.21, 95% CI 0.04–0.95, *p* = 0.03) and rs2289964C/T × rs11191979T/T (OR = 2.50, 95% CI 1.06–5.92, *p* = 0.05), along with three diplotypes—rs11191736C/C × rs187304410G/G (OR = 5.99, 95% CI 1.34–26.89, *p* = 0.01), rs34040810C/C × rs187304410G/G (OR = 3.91, 95% CI 1.09–14.05, *p* = 0.02), and rs34040810C/C × rs187304410G/A (OR = 0.24, 95% CI 0.06–0.98, *p* = 0.05)—were found to be associated with the risk of psoriasis risk in males and females, respectively. Nevertheless, none of these associations remained significant after applying the Benjamini–Hochberg procedure for multiple testing corrections.

Considering that glutathione S-transferases utilize glutathione for the detoxification of xenobiotics, it is essential to investigate the potential interactive effects of polymorphisms in the GSTO1 gene and the gene encoding the catalytic subunit of glutamate cysteine ligase (*GCLC*) on susceptibility to psoriasis. Genotyping data for six common polymorphisms of the *GCLC* gene in patients with psoriasis and controls were obtained from our previous study [39]. As shown in Supplementary Table S3, diplotypes *GSTO1* rs34040810C/C × *GCLC* rs648595G/G (OR = 0.70 95% CI 0.50–0.99, *p* = 0.04), *GSTO1* rs11191979T/T × *GCLC* rs542914C/C (OR = 1.44 1.02–2.02, *p* = 0.04), *GSTO1* rs11191979T/C × *GCLC* rs542914C/C (OR = 0.69 95% CI 0.48–0.99, *p* = 0.04), and *GSTO1* rs11191979T/C × *GCLC* rs648595G/G (OR = 0.55 95% CI 0.33–0.94, *p* = 0.03) were associated with the risk of psoriasis. These associations also did not survive after adjustment for multiple testing.

### 3.3. The Role of Gene–Gene and Gene–Environment Interactions in the Risk of Psoriasis

The comprehensive assessment of gene-by-gene interactions poses a challenge commonly known as the “curse of dimensionality”. This phenomenon arises in the context of epistatic analysis (epistasis is a genetic phenomenon whereby the impact of a mutation in a particular gene is influenced by the presence or absence of mutations in one or more additional genes), where the number of SNPs increases exponentially, significantly limiting the effectiveness of traditional parametric statistical methods [46,47,48]. In order to address this challenge, we employed the model-based multifactor dimensionality reduction approach, as outlined by Calle and colleagues [43], to assess the interactions between genes and between genes and environmental factors that are associated with susceptibility to psoriasis.

The distribution of environmental risk factors assessed in the study participants is presented in Table 4. The prevalence of alcohol abuse was higher among patients with psoriasis compared to healthy controls, both in the overall group and when stratified by sex (*p* < 0.0001). In addition, decreased consumption of fresh fruits and vegetables was associated with an increased risk of psoriasis in females (*p* = 7.1 × 10^−8^) but not in males (*p* = 0.07). No differences in cigarette smoking were observed between the case and control groups.

Second-, third-, and fourth-order G × G and G × E models, which include combinations of SNPs at the *GSTO1* and *GCLC* genes, along with the aforementioned risk factors, were analyzed to identify associations with the risk of psoriasis. Table 5 summarizes the number of mbmdr models that demonstrate significant associations between SNP × risk factor interactions and the risk of developing psoriasis. A total of 12 second-order, 68 third-order, and 239 fourth-order statistically significant (Pperm < 0.05) mbmdr models associated with the risk of psoriasis were established for the entire group. The primary findings indicated that the vast majority of models linking the risk of psoriasis were established due to gene–environment interactions, with alcohol abuse emerging as a significant risk factor. Notably, the highest number of gene–environment interactions was identified between alcohol abuse and the polymorphisms of the *GSTO1* and *GCLC* genes. Furthermore, many complex (third- and fourth-order) mbmdr models comprised cigarette smoking along with the interactions of gene polymorphisms and alcohol abuse.

Distinct characteristics of gene–environment interactions in males and females were identified. Notably, among the top five n-th-order mbmdr models, the risk factor of smoking was associated with 11 G × E interactions in females, while only one gene–smoking interaction (ALCOHOL × SMOKE × *GCLC* rs542914 × *GSTO1* rs11191979) was identified in males. As illustrated in Table 5, statistically significant differences between sexes were observed in both third-order and fourth-order models. In particular, the number of models involving ALCOHOL × *GCLC* (1 SNP) × *GSTO1* (1 SNP) was twice as high in males compared to females. The ALCOHOL × *GSTO1* (2 SNPs) × *GCLC* (1 SNP) model accounted for nearly 36% of the fourth-order models, with no corresponding model identified in females. In contrast, the number of ALCOHOL × SMOKE × *GCLC* (1 SNP) × *GSTO1* (1 SNP) and ALCOHOL × SMOKE × *GSTO1* (2 SNPs) mbmdr models was higher in females than in males. The top five n-th-order mbmdr G×E models associated with the risk of psoriasis in both males and females are presented in Table 6 and Table 7, respectively. Polymorphisms of the *GSTO1* gene were identified in 19 of the best mbmdr models for males and 14 models for females. Notably, the second-order mbmdr models for females are exclusively composed of polymorphisms in the *GSTO1* gene. In contrast, the models for males are influenced by both gene–environment interactions, incorporating three SNPs of *GSTO1* and two variants of *GCLC*. Additionally, SNPs of the *GCLC* gene were detected in nine models for males and five models for females.

## 4. Discussion

Glutathione S-transferases (GSTs) play a crucial role in detoxifying harmful xenobiotics, inactivating endogenous compounds resulting from oxidative stress, and synthesizing essential biological molecules such as leukotrienes, prostaglandins, testosterone, and progesterone [19]. In the context of xenobiotic biotransformation, GSTs utilize the antioxidant glutathione to facilitate the detoxification of both exogenous and endogenous compounds [19,38]. GSTs also exhibit a degree of antioxidant activity, particularly in relation to the inactivation of end products resulting from lipid peroxidation [49,50]. Hence, the association of these enzymes with oxidative stress can be attributed to the depletion of glutathione levels, which are utilized for neutralizing foreign chemical compounds. The omega class glutathione S-transferases (GSTO) are cytosolic enzymes that have been identified in various species, including humans [51]. In contrast to other classes of GSTs, which typically contain tyrosine or serine residues in their active sites, the active sites of GSTO enzymes feature an N-terminal cysteine residue that can bind to glutathione [52]. There is a growing body of evidence indicating that GSTO enzymes play a significant role in the detoxification of various exogenous stressors. In vitro studies indicate that human GSTO1 modulates the ryanodine receptor in the sarcoplasmic/endoplasmic reticulum, which is essential for Ca^2+^ release during excitation–contraction coupling in cardiac and skeletal muscles [53].

Furthermore, these studies indicated that human GSTO1 modulates the signaling pathway in c-Jun N-terminal kinase (JNK)-mediated apoptosis and activates interleukin-1β, a crucial mediator of inflammation [51]. Human GSTO1 has also been shown to regulate lipopolysaccharide-induced inflammatory responses in macrophages [54]. GSTO1 plays a critical role in redox homeostasis by influencing the glutathionylation and deglutathionylation of target proteins [51].

Oxidative stress is vital in psoriasis pathogenesis, prompting studies on the link between functionally significant glutathione S-transferase gene polymorphisms and disease risk. Certain studies indicate significant correlations between these variants and an increased susceptibility to psoriasis; however, other research has not found any associations with the risk of the disease [55,56]. The present study is the first to investigate whether polymorphisms in the *GSTO1* gene contribute to the risk of developing psoriasis. No statistically significant correlations were found between polymorphisms of the *GSTO1* gene and psoriasis risk when analyzing the overall case and control groups. The sex-stratified analysis revealed that two polymorphisms—specifically, rs11191736 in males and rs187304410 in females—are associated with the risk of psoriasis. Although the associations of psoriasis with the rs11191736 and rs187304410 polymorphisms could not be replicated in the overall population of the UK Biobank, 40 other SNPs in the *GSTO1* gene have been identified as nominally associated with the risk of psoriasis (Supplementary Table S1). These findings clearly highlight the significant role of *GSTO1* gene polymorphisms in psoriasis susceptibility. Analyses of haplotypes and diplotypes indicated that there were either no significant joint effects or only weak associations between the combinations of *GSTO1* genotypes and the risk of developing psoriasis. Nonetheless, bioinformatics modeling of the non-linear interactions between *GSTO1* and *GCLC* gene polymorphisms using the mbmdr method has revealed the existence of higher-order epistatic interactions between these genes, as well as with environmental risk factors, in relation to the risk of developing psoriasis. Moreover, the vast majority of mbmdr G x E models incorporated the combined effects of alcohol abuse and the polymorphisms of the *GSTO1* and *GCLC* genes on the risk of developing the disease. Research indicates that alcohol abuse significantly affects both the onset and progression of psoriasis [57,58]. Our recent study identified the joint effects of alcohol consumption and *GCLC* gene polymorphisms on psoriasis risk [39]. Apparently, these gene–environment interactions can be elucidated by the fact that, on one hand, the consumption of substantial amounts of alcohol is linked to the production of a significant number of reactive oxygen species and oxidative stress [59,60]. This process depletes the reserves of reduced glutathione and causes damage to liver tissue, where the majority of the body’s glutathione is synthesized [61,62]. On the other hand, the development of oxidative stress may be exacerbated by reduced glutathione synthesis resulting from decreased expression of the *GCLC* gene and/or increased utilization of reduced glutathione by the GSTO1 enzyme.

To understand the mechanisms by which polymorphisms are associated with the onset of psoriasis, it is crucial to analyze the functional annotation data of the *GSTO1* polymorphisms. SNP rs187304410, which has been associated with a reduced risk of psoriasis in females according to the GTEx portal (https://www.gtexportal.org/home/, accessed on 8 December 2024), correlates with alternative splicing events of the *GSTO1* and *GSTO2* genes in suprapubic and lower leg skin, as well as in cultured skin fibroblasts (https://www.gtexportal.org/home/, accessed on 8 December 2024). One of the alternative splicing sites is situated within intron 9 and is associated with a reduction in the expression of one GSTO1 isoform. Conversely, the other site, located in intron 7, is linked to an elevated production of a different isoform of the enzyme. It is well-established that alternative splicing can lead to the generation of multiple isoforms, some of which remain unknown [63]. To address the question regarding the properties and functions of the presumed GSTO1 isoforms with altered functional activity, it is essential to conduct experimental studies aimed at determining enzyme activity, substrate specificity, and other characteristics. In the blood, the polymorphism rs187304410 (allele A) is strongly associated with the expression levels of *GSTO2*, according to data from the eQTLGen consortium (https://www.eqtlgen.org/cis-eqtls.html, accessed on 8 December 2024). Other SNPs were found to be correlated with the expression levels of the *GSTO1* and *GSTO2* genes in the skin. The *GSTO1* and *GSTO2* genes are situated in proximity to one another on the same chromosomal segment, 10q25.1. Their transcriptional activity in the skin, specifically in foreskin fibroblast primary cells, seems to be regulated by shared enhancers found in the adjacent genomic region 104470800-104472000, as indicated by the Super-Enhancer database (http://www.licpathway.net/sedb/, accessed on 8 December 2024).

As mentioned above, GSTOs are involved in the biotransformation of xenobiotics and play a crucial role in protecting the skin from damage caused by environmental chemicals that penetrate both the superficial and deeper layers of the skin through transcutaneous and systemic routes [10,64]. Hence, the skin is one of the primary targets of environmental pollutants, and the disruption of the skin barrier due to inadequate xenobiotic biotransformation may potentially cause or exacerbate psoriasis. In particular, certain chemicals, especially drugs such as hydroxychloroquine [65], imiquimod [66], and lithium [67], have been identified as agents that can induce and exacerbate psoriasis. Per- and polyfluoroalkyl substances (PFAS), a class of synthetic chemicals extensively used in various consumer products, have been found to contribute to the risk of psoriasis [68]. Human exposure to PFAS primarily occurs through ingestion and inhalation, with the main sources being food, drinking water, and airborne dust. GSTO1 and GSTO2 are involved in the biotransformation of inorganic arsenic and play a crucial role in the reduction of monomethylarsonic acid, a highly toxic intermediate in the methylation process of inorganic arsenic [69,70]. Monomethylarsonic acid, a metabolite of arsenic, is the active ingredient in over 600 herbicides used in various agricultural products, including cotton, almonds, and oranges, as well as in landscaping applications [71]. GSTO2, like GSTO1, exhibits glutathione-dependent thiol transferase activity and shows significantly higher dehydroascorbate reductase activity—approximately 70 to 100 times greater than that of GSTO1 [70]. This enhanced activity may contribute to the regeneration of ascorbic acid, thereby providing protection against oxidative stress. This function of GSTO2 aligns with research findings indicating that ascorbic acid alleviates symptoms associated with skin lesions in psoriasis [37,72].

The present study revealed an intriguing finding regarding sexual dimorphism in the associations between psoriasis and single nucleotide polymorphisms in the *GSTO1* gene, both individually and in combination with *GCLC* variants and environmental risk factors. In females, the impact of polymorphisms in the *GSTO1* gene on the risk of psoriasis was more pronounced than that of the *GCLC* gene. In contrast, in males, the polymorphisms of both genes contributed nearly equally to the risk of developing the disease. These findings are not surprising, as genetic markers often demonstrate sexual dimorphism in their associations with various multifactorial diseases [73,74], including psoriasis [75,76]. The fundamental biological mechanisms that contribute to these sex differences are not yet fully elucidated. However, we believe that the sexual dimorphism observed in the associations between psoriasis and the SNPs of the GSTO1 and GCLC genes—both individually and in interaction with one another and environmental factors—can be attributed to several reasons. In our view, the most plausible explanation for the observed differences in SNP–disease associations between men and women is the influence of environmental risk factors. Specifically, alcohol abuse and a dietary deficiency in fresh fruits and vegetables have been linked to psoriasis, as supported by our data (Table 4) and corroborated by existing literature [57,58,59,77,78]. It is well established that the identified risk factors are associated with a reduction in endogenous glutathione. This reduction may occur due to its depletion from the toxic effects of ethanol [79] or insufficient synthesis resulting from a decreased intake of amino acid precursors of glutathione, which are found in high concentrations in unprocessed, plant-based foods [80]. The effects of functionally inferior alleles of the polymorphisms in the studied genes appear to exacerbate the deficiency of glutathione in psoriasis. This deficiency arises from insufficient synthesis involving glutamate cysteine ligase and/or excessive utilization by *GSTO1* for the detoxification of xenobiotics. Thus, the unidirectional influence of environmental factors, combined with polymorphisms in *GSTO1* and *GCLC*, seems to disrupt glutathione metabolism. This disruption may lead to conditions that promote oxidative stress and contribute to the onset of psoriasis. Another possible explanation for the sexual dimorphism observed in the associations between *GSTO1* and *GCLC* polymorphisms and psoriasis is that women generally exhibit greater sensitivity to the toxic effects of chemical pollutants compared to men, as evidenced by some studies [15,81]. It is noteworthy that this phenomenon also appears to manifest at the level of associations between polymorphisms of genes encoding xenobiotic biotransformation enzymes, one of which is glutathione S-transferase omega 1. In other words, the genes we studied that encode biotransformation enzymes, similar to other genes involved in xenobiotic metabolism, may be differentially regulated in men and women, a fact that has been well established [82,83].

Our study has both strengths and limitations. This investigation is the first to explore the associations between *GSTO1* gene polymorphisms and the risk of psoriasis. It is also one of the largest studies conducted on GST gene polymorphisms and their susceptibility to psoriasis. The application of the mbmdr methods enabled the identification of epistatic interactions between the polymorphisms of the *GSTO1* gene and variants of *GCLC* in determining the risk of psoriasis. Given that our research is a pilot study, it is essential to conduct further investigations in independent populations to validate the associations identified between *GSTO1* gene polymorphisms and the risk of psoriasis. Furthermore, as our study examined a relatively limited number of single nucleotide polymorphisms, it is advisable for future genetic association studies to explore the relationship between psoriasis and a broader array of polymorphisms, including those that have demonstrated associations with psoriasis in the UK Biobank cohorts (as detailed in the Supplementary Table S1). Additionally, since the polymorphisms analyzed in the *GSTO1* gene are located in noncoding regions, any phenotypic implications should be approached with caution, as no assessments of gene expression in skin biopsies from the study participants have been conducted. Finally, other environmental factors that were not analyzed in the present study may also influence the risk of developing psoriasis.

## 5. Conclusions

**Figure 1:**
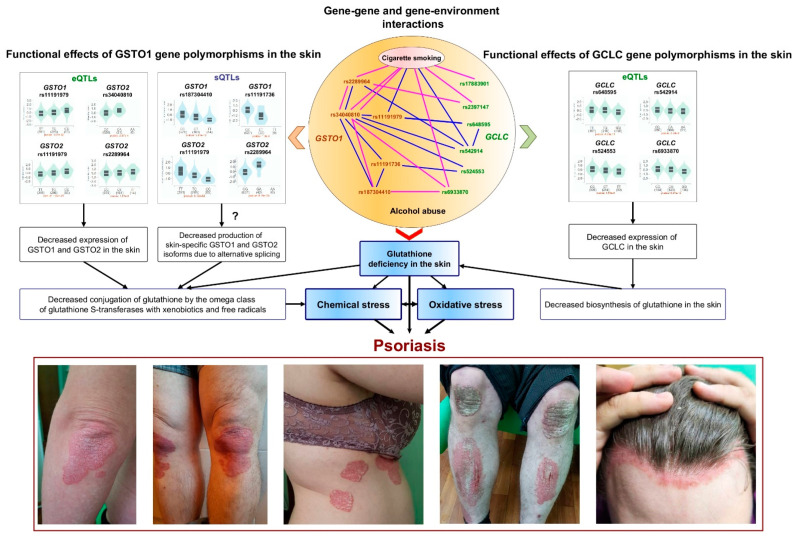
Proposed mechanisms for the involvement of *GSTO1* and *GCLC* gene polymorphisms in the pathogenesis of psoriasis.

The present study demonstrates, for the first time, that polymorphisms in the gene encoding glutathione S-transferase omega 1 serve as novel genetic markers for susceptibility to psoriasis. Notably, various polymorphisms in the *GSTO1* gene exhibited synergistic effects on the risk of psoriasis and contributed to disease susceptibility in a sex-dependent manner. Additionally, we identified joint effects on disease risk for polymorphisms in the *GSTO1* gene and the catalytic subunit of the glutamate cysteine ligase gene, which encodes the first enzyme in the cellular glutathione biosynthetic pathway. Furthermore, we observed that environmental risk factors, such as alcohol abuse and cigarette smoking, also exhibited joint effects with *GSTO1* and *GCLC* polymorphisms on the risk of developing psoriasis. The results of the study provide new evidence confirming that psoriasis is a typical multifactorial disease, resulting from a complex interaction of genetic and environmental factors. In addition, this study emphasizes the crucial role of impaired glutathione detoxification mechanisms in the molecular pathogenesis of psoriasis. Potential mechanisms by which *GSTO1* gene polymorphisms may contribute to the development of psoriasis are summarized in Figure 1. The phenotypic effects associated with *GSTO1* gene polymorphisms are likely mediated by alterations—specifically reductions—in the expression levels of the *GSTO1* and *GSTO2* genes, as well as other adjacent genes that may be regulated by shared enhancers located within the genomic region 10q25.1. Polymorphisms in *GSTO1* and *GSTO2* are known to be sites for alternative splicing of these genes in the skin. This may lead to the production of functionally distinct enzyme isoforms with varying activities concerning the conjugation of cutaneous xenobiotics and free radicals with glutathione. Increased consumption of glutathione by glutathione S-transferases and glutathione peroxidases, which neutralize free radicals and xenobiotics, along with the harmful effects of alcohol, smoking, and other external factors, contributes to the depletion of glutathione, in addition to its reduced synthesis by glutamate cysteine ligase. Consequently, these disorders lead to the accumulation of free radicals and toxic substances, thereby initiating oxidative stress and causing damage to skin tissue. It is unequivocal that oxidative stress acts as a mechanistic pathway in the pathogenesis of psoriasis and represents a fundamental mechanism underlying the detrimental effects of environmental chemicals [9]. Gaining deeper insights into how the polymorphisms of the *GSTO1* gene interact with other genes associated with glutathione metabolism could pave the way for innovative, evidence-based strategies for treating and preventing psoriasis.

The central section of the figure provides a schematic representation of the interactions between single nucleotide polymorphisms and their relationships with environmental factors, specifically alcohol abuse and cigarette smoking. These factors are the most significantly correlated with the risk of psoriasis, as determined by the top five n-th-order mbmdr models. The blue lines in the central section represent interactions among males, while the pink lines indicate interactions among females. The left and right sections of the figure display violin plots of expression quantitative trait loci (eQTLs) and splicing quantitative trait loci (sQTLs) for the studied SNPs obtained from the GTEx portal (https://www.gtexportal.org/home/, accessed on 8 December 2024). Below the plots are photographs of patients with psoriasis from our own collection. Comments regarding the figure are provided in the main text of the article.

## Figures and Tables

**Table 1:** A summary of associations between *GSTO1* gene polymorphisms and psoriasis risk in the entire group and sex-stratified groups.

SNP ID	Minor Allele	N	Permutation *p*-Values (P_perm_) Estimated for Genetic Models of SNP–Disease Associations *			
			Allelic	Additive	Dominant	Recessive
Entire group						
rs11191736	T	901	0.26	0.22	0.19	NA
rs34040810	A	944	0.45	0.29	0.39	NA
rs2289964	T	941	0.86	1.00	0.78	0.86
rs11191979	C	930	0.59	1.00	0.67	0.73
rs187304410	A	944	0.86	0.31	0.52	NA
Males						
rs11191736	T	467	**0.02**	NA	NA	NA
rs34040810	A	486	0.34	0.39	0.64	NA
rs2289964	T	486	0.78	0.64	0.55	0.59
rs11191979	C	479	0.71	0.67	0.52	0.59
rs187304410	A	486	0.46	0.31	0.29	NA
Females						
rs11191736	T	434	0.25	NA	NA	NA
rs34040810	A	458	0.86	0.63	0.55	NA
rs2289964	T	455	0.33	0.33	0.29	NA
rs11191979	C	451	0.86	0.86	1.00	1.00
rs187304410	A	458	**0.02**	**0.012**	**0.011**	NA

* Significance of SNP–disease associations was assessed by adaptive permutations using PLINK software v.1.9. NA, not available: this indicates that there were no individuals with the specified genotypes required for the calculation of the corresponding genetic model. Bold depicts statistically significant *p*-values.

**Table 2:** Genotype and allele frequencies of the *GSTO1* gene and their associations with psoriasis risk in the entire group and sex-stratified groups.

SNP	Genotype/ Allele	Healthy Controls ^1^		Patients with Psoriasis ^1^		OR ^2^ (95% CI)	P_perm_^3^
		n	%	n	%		
Entire group							
rs11191736	C/C	454	99.3	438	98.6	2.07 (0.52–8.34)	0.19 ^D^
	C/T	3	0.7	6	1.4		
	T/T	0	0.0	0	0.0		
	T	3	0.3	6	0.7	2.07 (0.52–8.29)	0.26
rs34040810	C/C	465	98.9	466	98.3	1.60 (0.52–4.92)	0.29 ^A^
	C/A	5	1.1	8	1.7		
	A/A	0	0.0	0	0.0		
	A	5	0.5	8	0.8	1.59 (0.52–4.88)	0.45
rs2289964	C/C	360	76.6	365	77.5	0.95 (0.70–1.29)	0.78 ^D^
	C/T	103	21.9	99	21.0		
	T/T	7	1.5	7	1.5		
	T	117	12.4	113	12.0	0.96 (0.73–1.26)	0.86
rs11191979	T/T	236	51.3	248	52.8	0.94 (0.73–1.22)	0.53 ^D^
	T/C	192	41.7	183	38.9		
	C/C	32	7.0	39	8.3		
	C	256	27.8	261	27.8	1.00 (0.81–1.22)	0.59
rs187304410	G/G	451	96.0	459	96.8	0.78 (0.39–1.55)	0.31 ^A^
	G/A	19	4.0	15	3.2		
	A/A	0	0.0	0	0.0		
	A	19	2.0	15	1.6	0.78 (0.39–1.54)	0.86
Males							
rs11191736	C/C	226	100.0	235	97.5	NA	NA
	C/T	0	0.0	6	2.5		
	T/T	0	0.0	0	0.0		
	T	0	0.0	6	1.2	NA	**0.017**
rs34040810	C/C	231	98.7	245	97.2	2.20 (0.56–8.6)	0.39 ^A^
	C/A	3	1.3	7	2.8		
	A/A	0	0.0	0	0.0		
	A	3	0.6	7	1.4	2.18 (0.56–8.49)	0.34
rs2289964	C/C	185	79.1	192	76.2	1.18 (0.77–1.81)	0.55 ^D^
	C/T	42	17.9	54	21.4		
	T/T	7	3.0	6	2.4		
	T	56	12.0	66	13.1	1.11 (0.76–1.62)	0.78
rs11191979	T/T	111	48.5	129	51.6	0.88 (0.62–1.26)	0.52 ^D^
	T/C	100	43.7	98	39.2		
	C/C	18	7.9	23	9.2		
	C	136	29.7	144	28.8	0.96 (0.72–1.27)	0.71
rs187304410	G/G	226	96.6	239	94.8	1.54 (0.63–3.78)	0.29 ^D^
	G/A	8	3.4	13	5.2		
	A/A	0	0.0	0	0.0		
	A	8	1.7	13	2.6	1.52 (0.63–3.71)	0.46
Females							
rs11191736	C/C	228	98.7	203	100.0	NA	NA
	C/T	3	1.3	0	0.0		
	T/T	0	0.0	0	0.0		
	T	3	0.6	0	0.0	NA	0.25
rs34040810	C/C	234	99.2	221	99.5	0.53 (0.05–5.88)	0.55 ^D^
	C/A	2	0.8	1	0.5		
	A/A	0	0.0	0	0.0		
	A	2	0.4	1	0.2	0.53 (0.05–5.87)	0.86
rs2289964	C/C	175	74.2	173	79.0	0.76 (0.49–1.18)	0.29 ^D^
	C/T	61	25.8	45	20.5		
	T/T	0	0.0	1	0.5		
	T	61	12.9	47	10.7	0.81 (0.54–1.21)	0.33
rs11191979	T/T	125	54.1	119	54.1	1.03 (0.77–1.39)	0.86 ^A^
	T/C	92	39.8	85	38.6		
	C/C	14	6.1	16	7.3		
	C	120	26.0	117	26.6	1.03 (0.77–1.39)	0.86
rs187304410	G/G	225	95.3	220	99.1	**0.19 (0.04–0.85)**	**0.012** ^D^
	G/A	11	4.7	2	0.9		
	A/A	0	0.0	0	0.0		
	A	11	2.3	2	0.5	**0.19 (0.04–0.86)**	**0.02**

The table shows the best genetic models for SNP–disease associations. ^1^ Absolute number and percentage of individuals and chromosomes with a particular genotype and allele, respectively. ^2^ Odds ratio with 95% confidence intervals (crude analysis) estimated for the best SNP–disease association model. ^3^ *p*-Value estimated for the best SNP–disease association model through adaptive permutations. Superscripts denote the models: D, dominant; A, additive. Bold depicts statistically significant *p*-values and odds ratios. NA, not available: this indicates that there were no individuals with the specified genotypes required for the calculation of the corresponding genetic model.

**Table 3:** Haplotypes of the *GSTO1* gene and their association with psoriasis risk in the entire group and sex-stratified groups.

Haplotypes	SNP					Patients with Psoriasis	Healthy Controls	Chi-Square	*p*-Value
	rs11191736	rs34040810	rs2289964	rs11191979	rs187304410				
Entire group									
H1	C	C	C	T	G	0.671	0.672	0.005	0.999
H2	C	C	C	C	G	0.200	0.190	0.314	0.972
H3	C	C	T	C	G	0.076	0.085	0.589	0.951
H4	C	C	T	T	G	0.032	0.030	0.070	0.993
Rare	-	-	-	-	-	0.021	0.023	-	-
Males									
H1	C	C	C	T	G	0.645	0.657	0.118	1.000
H2	C	C	C	C	G	0.208	0.215	0.097	1.000
H3	C	C	T	C	G	0.076	0.084	0.089	1.000
H4	C	C	T	T	G	0.036	0.026	0.436	0.953
Rare	-	-	-	-	-	0,035	0,018	-	-
Females									
H1	C	C	C	T	G	0.701	0.687	0.233	1.000
H2	C	C	C	C	G	0.187	0.165	0.733	1.000
H3	C	C	T	C	G	0.077	0.089	0.438	1.000
H4	C	C	T	T	G	0.028	0.032	0.094	1.000
Rare	-	-	-	-	-	0.007	0.027	-	-

Estimation of haplotype frequencies and significance of haplotype–disease associations was performed using Haploview software, v.4.2.

**Table 4:** Environmental risk factors among study participants.

Environmental Risk Factors	Patients with Psoriasis n (%)	Healthy Controls n (%)	*p*-Value *
Overall group			
Smokers (ever/never) ^1^	168 (35.4)	148 (31.5)	0.20
Alcohol abusers ^2^	105 (21.2)	7 (3.2)	**<0.0001**
Decreased consumption of fresh fruits and vegetables ^3^	183 (38.6)	68 (30.0)	**0.03**
Males			
Smokers (ever/never)	120 (47.6)	106 (45.3)	0.61
Alcohol abusers	74 (29.4)	6 (5.2)	**3.6 × 10^−7^**
Decreased consumption of fresh fruits and vegetables	93 (36.9)	56 (46.7)	0.07
Females			
Smokers (ever/never)	48 (21.6)	42 (17.8)	0.30
Alcohol abusers	31 (14.0)	1 (1.0)	**0.0005**
Decreased consumption of fresh fruits and vegetables	90 (40.5)	12 (11.2)	**7.1 × 10^−8^**

^1^ Data on smoking status were available for 474 patients with psoriasis and 470 individuals in the control group. ^2^ Data on alcohol consumption were available for 474 patients with psoriasis and 220 individuals in the control group. ^3^ Data on fresh fruit and vegetable intake were available for 474 patients with psoriasis and 227 individuals in the control group. * Bold indicates a statistically significant *p*-value.

**Table 5:** Summary of the number of mbmdr models of SNP × risk factor interactions significantly associated with psoriasis risk.

mbmdr Models of SNP × Risk Factor Interactions	Entire Group ^1^		Males ^1^		Females ^1^	
	N	%	N	%	N	%
Second-order models	N = 12		N = 16		N = 13	
ALCOHOL × *GSTO1* (1 SNP)	5	41.7	5	31.3	5	38.5
ALCOHOL × *GCLC* (1 SNP)	6	50.0	6	37.5	6	46.2
ALCOHOL × SMOKE	1	8.3	1	6.3	1	7.7
*GSTO1* (1 SNP) × *GCLC* (1 SNP)	0	0.0	4	25.0	0	0.0
*GSTO1* (1 SNP) × *GSTO1* (1 SNP)	0	0.0	0	0.0	1	7.7
Third-order models	N = 68		N = 83		N = 28	
ALCOHOL × *GSTO1* (1 SNP) × *GSTO1* (1 SNP)	10	14.7	10	12.0	8	28.6
ALCOHOL × *GCLC* (1 SNP) × *GSTO1* (1 SNP) *	30	44.1	45	54.2	8	28.6
ALCOHOL × *GCLC* (1 SNP) × *GCLC* (1 SNP)	15	22.1	15	18.1	1	3.6
ALCOHOL × SMOKE × *GSTO1* (1 SNP)	5	7.4	5	6.0	5	17.9
ALCOHOL × SMOKE × *GCLC* (1 SNP)	6	8.8	6	7.2	6	21.4
Other models	2	2.9	2	2.4	0	0.0
Fourth-order models	N = 239		N = 148		N = 19	
ALCOHOL × *GSTO1* (3 SNPs)	10	4.2	9	6.1	0	0.0
ALCOHOL × *GSTO1* (2 SNPs) × *GCLC* (1 SNP) **	60	25.1	53	35.8	0	0.0
ALCOHOL × *GCLC* (2 SNPs) × *GSTO1* (1 SNP)	95	39.7	6	4.1	1	5.3
ALCOHOL × *GCLC* (3 SNPs)	20	8.4	14	9.5	0	0.0
ALCOHOL × SMOKE × *GCLC* (2 SNPs)	10	4.2	12	8.1	1	5.3
ALCOHOL × SMOKE × *GCLC* (1 SNP) × *GSTO1* (1 SNP) **	30	12.6	36	24.3	12	63.2
ALCOHOL × SMOKE × *GSTO1* (2 SNPs) *	10	4.2	10	6.8	5	26.3
Other models	4	1.7	8	5.4	0	0.0

^1^ The absolute number and percentage of a specific mbmdr model within a study group. Asterisks indicate that the mbmdr models demonstrated quantitative differences between males and females (* *p* < 0.05, ** *p* < 0.01). ALCOHOL, alcohol abuse; SMOKE, cigarette smoking.

**Table 6:** The best *n*-th-order mbmdr models of SNP × risk factor interactions significantly associated with psoriasis risk in males.

	mbmdr Models of SNP × Risk Factor Interactions	NH	β-H	WH	NL	β-L	WL	P_perm_
Second-order models								
1	ALCOHOL × *GSTO1* rs34040810	2	0.322	34.87	1	−0.322	34.87	**<0.0001**
2	ALCOHOL × *GSTO1* rs11191736	1	0.299	26.12	1	−0.313	30.68	**<0.0001**
3	ALCOHOL × *GSTO1* rs187304410	1	0.300	27.14	1	−0.292	29.57	**<0.0001**
4	ALCOHOL × *GCLC* rs648595	3	0.308	29.57	1	−0.259	14.33	**<0.0001**
5	ALCOHOL × *GCLC* rs542914	3	0.308	29.57	2	−0.183	14.68	**<0.0001**
Third-order models								
1	ALCOHOL × *GCLC* rs524553 × *GSTO1* rs34040810	2	0.297	25.88	3	−0.322	34.30	**<0.0001**
2	ALCOHOL × *GCLC* rs542914 × *GSTO1* rs34040810	4	0.322	34.30	2	−0.203	18.31	**<0.0001**
3	ALCOHOL × *GSTO1* rs34040810 × *GSTO1* rs11191736	1	0.299	26.12	1	−0.323	33.91	**<0.0001**
4	ALCOHOL × *GSTO1* rs11191979 × *GSTO1* rs34040810	4	0.317	33.31	2	−0.267	27.36	**<0.0001**
5	ALCOHOL × *GSTO1* rs2289964 × *GSTO1* rs34040810	3	0.316	32.45	1	−0.172	12.53	**<0.0001**
Fourth-order models								
1	ALCOHOL × *GCLC* rs648595 × *GCLC* rs542914 × *GSTO1* rs11191979	4	0.301	16.69	6	−0.303	38.90	**<0.0001**
2	ALCOHOL × *GCLC* rs524553 × *GSTO1* rs34040810 × *GSTO1* rs11191736	2	0.288	22.76	3	−0.322	33.34	**<0.0001**
3	ALCOHOL × *GCLC* rs542914 × *GSTO1* rs2289964 × *GSTO1* rs34040810	5	0.313	29.13	3	−0.167	11.98	**<0.0001**
4	ALCOHOL × *GSTO1* rs187304410 × *GSTO1* rs34040810 × *GSTO1* rs11191736	1	0.292	23.98	1	−0.292	28.65	**<0.0001**
5	ALCOHOL × *SMOKE* × *GCLC* rs542914 × *GSTO1* rs11191979	5	0.291	26.44	4	−0.279	28.03	**<0.0001**

NH—number of interacting high-risk variables (SNP and risk factors); β-H—regression coefficient for high-risk interactions identified in step 2 of the analysis. WH—Wald statistics for high-risk interactions; NL is the number of interacting low-risk variables; β-L—regression coefficient for low-risk interactions identified in step 2 of the analysis; WL—Wald statistics for low-risk interactions. P_perm_—permutation significance levels for the models associated with psoriasis risk. Bold is indicates a statistically significant *p*-value.

**Table 7:** The best *n*-th-order mbmdr models of SNP × risk factor interactions significantly associated with psoriasis risk in females.

	mbmdr Models of SNP × Risk Factor Interactions	NH	β-H	WH	NL	β-L	WL	P_perm_
Second-order models								
1	ALCOHOL × SMOKE	3	0.285	22.22	1	−0.285	22.22	**<0.0001**
2	ALCOHOL × *GSTO1* rs187304410	1	0.319	14.02	2	−0.319	14.02	**<0.0001**
3	ALCOHOL × *GSTO1* rs34040810	1	0.317	13.42	1	−0.288	11.62	**<0.0001**
4	ALCOHOL × *GSTO1* rs11191736	1	0.327	12.65	1	−0.291	10.29	**<0.0001**
5	ALCOHOL × *GSTO1* rs2289964	2	0.323	14.27	1	−0.159	6.21	**<0.0001**
Third-order models								
1	ALCOHOL × SMOKE × *GSTO1* rs2289964	3	0.263	15.51	2	−0.289	22.82	**<0.0001**
2	ALCOHOL × SMOKE × *GSTO1* rs187304410	3	0.285	22.22	2	−0.285	22.22	**<0.0001**
3	ALCOHOL × SMOKE × *GSTO1* rs34040810	3	0.282	21.52	1	−0.270	20.04	**<0.0001**
4	ALCOHOL × SMOKE × *GCLC* rs17883901	3	0.261	16.24	2	−0.187	10.81	**<0.0001**
5	ALCOHOL × SMOKE × *GCLC* rs542914	3	0.320	14.56	1	−0.236	8.39	**<0.0001**
Fourth-order models								
1	ALCOHOL × SMOKE × *GCLC* rs2397147 × *GSTO1* rs2289964	2	0.292	8.24	3	−0.238	22.18	**<0.0001**
2	ALCOHOL × SMOKE × *GSTO1* rs187304410 × *GSTO1* rs34040810	3	0.282	21.52	2	−0.270	20.04	**<0.0001**
3	ALCOHOL × SMOKE × *GCLC* rs6933870 × *GSTO1* rs187304410	4	0.342	21.24	2	−0.166	9.87	**<0.0001**
4	ALCOHOL × SMOKE × *GSTO1* rs2289964 × *GSTO1* rs34040810	3	0.263	15.51	2	−0.274	20.61	**<0.0001**
5	ALCOHOL × SMOKE × *GCLC* rs6933870 × *GSTO1* rs34040810	4	0.340	20.56	1	−0.165	9.84	**<0.0001**

NH—number of interacting high-risk variables (SNP and risk factors); β-H—regression coefficient for high-risk interactions identified in step 2 of the analysis. WH—Wald statistics for high-risk interactions; NL is the number of interacting low-risk variables; β-L—regression coefficient for low-risk interactions identified in step 2 of the analysis; WL—Wald statistics for low-risk interactions. P_perm_—permutation significance levels for the models associated with psoriasis risk. Bold is indicates a statistically significant *p*-value.

## Data Availability

The data presented in this study are available upon reasonable request from the corresponding author due to sensitivity concerns.

## Supplementary Materials

The following supporting information can be downloaded at: https://www.mdpi.com/article/10.3390/jox15020060/s1, Supplementary Table S1: Associations between the polymorphisms of the *GSTO1* gene and psoriasis risk in a population of the UK Biobank; Supplementary Table S2: *GSTO1* genotype combinations and their associations with psoriasis risk; Supplementary Table S3: *GSTO1* by *GCLC* genotype combinations and their associations with psoriasis risk in the entire group; Supplementary Table S4: The best n-th-order mbmdr models of SNP × risk factor interactions significantly associated with psoriasis risk in the entire group.
