# Supplementary material for: Genetic Interactions of Phase II Xenobiotic-Metabolizing Enzymes GSTO1 and GCLC in Relation to Alcohol Abuse and Psoriasis Risk
Source: J Xenobiot. 2025 Apr 20;15(2):60. doi: 10.3390/jox15020060 (PMC12028938; doi:10.3390/jox15020060)
Supplement: Supplementary file 1 [file jox-15-00060-s001.zip › jox-3420050-supplementary.pdf]

Supplementary Table S1 Associations between the polymorphisms of the *GSTO1* gene and psoriasis risk in a population of the UK Biobank[1]

N	Variant	Position	Eff, Allele	Psoriasis phenotype[2]	Beta	P-value	MAF	HWE
1	rs45579835	106059390	T	psoriasis	2.6E-03	0.006	0.015	0.67
2	rs10883979	105957714	A	psoriasis	-1.3E-03	0.007	0.053	0.04
3	rs10883986	105992144	A	psoriasis	1.3E-03	0.008	0.053	0.03
4	rs71473514	105970740	A	psoriasis	1.3E-03	0.008	0.053	0.03
5	rs11191960	105990647	A	psoriasis	1.3E-03	0.008	0.053	0.04
6	rs10883988	105994128	C	psoriasis	1.3E-03	0.008	0.053	0.03
7	rs11191947	105963553	G	psoriasis	1.3E-03	0.008	0.053	0.03
8	rs10883978	105950516	T	psoriasis	1.3E-03	0.008	0.053	0.03
9	rs11191946	105962291	A	psoriasis	1.3E-03	0.008	0.053	0.03
10	rs10883984	105982620	C	psoriasis	1.3E-03	0.008	0.053	0.03
11	rs10883980	105958440	T	psoriasis	1.3E-03	0.009	0.053	0.03
12	rs10883983	105977766	C	psoriasis	1.3E-03	0.009	0.053	0.03
13	rs10883985	105982792	C	psoriasis	1.3E-03	0.009	0.053	0.03
14	rs10883989	106000421	T	psoriasis	1.3E-03	0.009	0.053	0.03
15	rs11191952	105979638	C	psoriasis	1.3E-03	0.009	0.053	0.03
16	rs72825847	106043560	G	psoriasis	3.6E-03	0.009	0.006	0.06
17	rs72825843	106036159	G	psoriasis	3.6E-03	0.009	0.006	0.06
18	rs146972819	106001422	A	psoriasis	3.6E-03	0.009	0.007	0.42
19	rs72825838	106015458	G	psoriasis	3.6E-03	0.009	0.006	0.06
20	rs7907367	105966362	T	psoriasis	1.3E-03	0.009	0.053	0.03
21	rs958118	105985410	C	psoriasis	1.3E-03	0.009	0.053	0.03
22	rs480185	105983609	T	psoriasis	1.3E-03	0.009	0.053	0.03
23	rs522148	105993530	A	psoriasis	1.3E-03	0.010	0.053	0.03
24	rs1086314	105984238	A	psoriasis	1.3E-03	0.010	0.053	0.03
25	rs11191961	105993137	T	psoriasis	1.3E-03	0.010	0.053	0.03
26	rs74398245	106064814	A	psoriasis	-2.9E-03	0.010	0.010	0.93
27	rs11191962	105993301	C	psoriasis	1.3E-03	0.010	0.053	0.03
28	rs34973112	105991388	T	psoriasis	1.2E-03	0.011	0.054	0.03
29	rs78961630	106007591	G	psoriasis	1.7E-03	0.012	0.026	0.08
30	rs114174365	106075500	G	psoriasis	-3.5E-03	0.012	0.006	0.10
31	rs117010634	105993961	A	psoriasis	1.5E-03	0.015	0.032	0.20
32	rs147836412	106056280	A	psoriasis	-1.6E-03	0.018	0.030	0.22
33	rs45468393	106059553	G	psoriasis	3.8E-03	0.020	0.004	0.25
34	rs191009296	105993822	C	psoriasis	4.4E-03	0.022	0.004	0.64
35	rs576542861	106035215	A	psoriasis	-4.3E-03	0.024	0.004	0.38
36	rs535554028	106044677	T	psoriasis	5.7E-03	0.025	0.002	1.00
37	rs117946494	105999115	T	psoriasis	-2.2E-03	0.038	0.011	0.36
38	rs41291844	105967573	C	psoriasis	3.5E-03	0.040	0.004	0.41
39	rs17883344	106022869	T	psoriasis	-1.6E-03	0.047	0.019	0.96
40	rs74533873	105947657	C	psoriasis	2.0E-03	0.050	0.012	0.20

[1] The calculations were obtained from the Gene ATLAS web site (http://geneatlas.roslin.ed.ac.uk/). accessed by 13.08.2024. [2] "Psoriasis" phenotype investigated in a cohort of 5175 cases and 447089 controls; MAF. minor allele frequency; HWE. Hardy-Weinberg equilibrium P-value.

Supplementary Table S2 *GSTO1* genotype combinations and their associations with psoriasis risk

Genotype combination	Patients		Controls		OR (95% CI)[3]	*P*-value
	n[1]	%[2]	n[1]	%[2]		
Entire groups						
rs11191736C/C × rs34040810C/C	434	97.7	452	98.9	0.48 (0.16-1.42)	0.17
rs11191736C/C × rs34040810C/A	4	0.9	2	0.4	1.86 (0.39-8.78)	0.66
rs11191736C/T × rs34040810C/C	4	0.9	1	0.2	3.11 (0.49-19.82)	0.35
rs11191736C/T × rs34040810C/A	2	0.5	2	0.4	1.03 (0.18-5.97)	0.64
rs11191736C/C × rs2289964C/C	340	77.1	353	77.2	0.99 (0.73-1.35)	0.96
rs11191736C/C × rs2289964C/T	90	20.4	95	20.8	0.98 (0.71-1.35)	0.89
rs11191736C/C × rs2289964T/T	5	1.1	6	1.3	0.88 (0.28-2.75)	0.95
rs11191736C/T × rs2289964C/C	3	0.7	1	0.2	2.43 (0.36-16.52)	0.59
rs11191736C/T × rs2289964C/T	2	0.5	2	0.4	1.04 (0.18-6.01)	0.64
rs11191736C/T × rs2289964T/T	1	0.2	0	0.0	3.12 (0.13-76.69)	0.99
rs11191736C/C × rs11191979T/T	233	52.5	228	51.0	1.06 (0.82-1.38)	0.66
rs11191736C/C × rs11191979T/C	172	38.7	187	41.8	0.88 (0.67-1.15)	0.35
rs11191736C/C × rs11191979C/C	33	7.4	29	6.5	1.16 (0.69-1.94)	0.58
rs11191736C/T × rs11191979T/T	2	0.5	2	0.4	1.01 (0.17-5.84)	0.62
rs11191736C/T × rs11191979T/C	4	0.9	1	0.2	3.04 (0.48-19.39)	0.37
rs11191736C/C × rs187304410G/G	427	96.2	437	95.6	1.15 (0.59-2.22)	0.68
rs11191736C/C × rs187304410G/A	11	2.5	17	3.7	0.66 (0.30-1.42)	0.28
rs11191736C/T × rs187304410G/G	3	0.7	2	0.4	1.44 (0.28-7.36)	0.97
rs11191736C/T × rs187304410G/A	3	0.7	1	0.2	2.41 (0.35-16.41)	0.60
rs34040810C/C × rs2289964C/C	365	77.5	360	76.6	1.05 (0.78-1.43)	0.74
rs34040810C/C × rs2289964C/T	91	19.3	100	21.3	0.89 (0.64-1.22)	0.46
rs34040810C/C × rs2289964T/T	7	1.5	5	1.1	1.37 (0.45-4.14)	0.77
rs34040810C/A × rs2289964C/T	8	1.7	3	0.6	2.45 (0.70-8.56)	0.23
rs34040810C/A × rs2289964T/T	0	0.0	2	0.4	0.20 (0.01-4.15)	0.48
rs34040810C/C × rs11191979T/T	241	51.3	233	50.7	1.03 (0.79-1.33)	0.85
rs34040810C/C × rs11191979T/C	182	38.7	190	41.3	0.90 (0.69-1.17)	0.42
rs34040810C/C × rs11191979T/T	39	8.3	32	7.0	1.21 (0.74-1.97)	0.44
rs34040810C/A × rs11191979T/T	7	1.5	3	0.7	2.12 (0.59-7.57)	0.36
rs34040810C/A × rs11191979T/C	1	0.2	2	0.4	0.59 (0.08-4.45)	0.99
rs34040810C/C × rs187304410G/G	456	96.2	449	95.5	1.18 (0.62-2.25)	0.60
rs34040810C/C × rs187304410G/A	10	2.1	16	3.4	0.61 (0.27-1.36)	0.22
rs34040810C/A × rs187304410G/G	3	0.6	2	0.4	1.39 (0.27-7.09)	0.99
rs34040810C/A × rs187304410G/A	5	1.1	3	0.6	1.56 (0.41-6.01)	0.73
rs2289964C/C × rs11191979T/T	216	46.3	212	46.1	1.01 (0.78-1.30)	0.96
rs2289964C/C × rs11191979T/C	121	25.9	129	28.0	0.90 (0.67-1.20)	0.46
rs2289964C/C × rs11191979C/C	24	5.1	14	3.0	1.73 (0.88-3.38)	0.11
rs2289964C/T × rs11191979T/T	30	6.4	21	4.6	1.44 (0.81-2.55)	0.21
rs2289964C/T × rs11191979T/C	57	12.2	62	13.5	0.89 (0.61-1.31)	0.56
rs2289964C/T × rs11191979T/T	12	2.6	15	3.3	0.78 (0.36-1.69)	0.53
rs2289964T/T × rs11191979T/T	0	0.0	3	0.7	1.01 (0.01-2.71)	0.96
rs2289964T/T × rs11191979T/C	4	0.9	1	0.2	0.90 (0.47-18.96)	0.46
rs2289964T/T × rs11191979T/T	3	0.6	3	0.7	1.73 (0.22-4.36)	0.11
rs2289964C/C × rs187304410G/G	358	76.0	350	74.5	1.09 (0.81-1.46)	0.58
rs2289964C/C × rs187304410G/A	7	1.5	10	2.1	0.71 (0.27-1.82)	0.62
rs2289964C/T × rs187304410G/G	92	19.5	95	20.2	0.96 (0.70-1.32)	0.79
rs2289964C/T × rs187304410G/A	7	1.5	8	1.7	0.88 (0.33-2.37)	1.00
rs2289964T/T × rs187304410G/G	6	1.3	6	1.3	1.00 (0.33-2.98)	0.77
rs2289964T/T × rs187304410G/A	1	0.2	1	0.2	1.00 (0.10-9.63)	0.48

rs11191979T/T × rs187304410G/G	236	50.2	223	48.5	1.07 (0.83-1.39)	0.60
rs11191979T/T × rs187304410G/A	12	2.6	13	2.8	0.90 (0.41-2.00)	0.80
rs11191979T/C × rs187304410G/G	180	38.3	188	40.9	0.90 (0.69-1.17)	0.42
rs11191979T/C × rs187304410G/A	3	0.6	4	0.9	0.76 (0.19-3.09)	0.98
rs11191979C/C × rs187304410G/G	39	8.3	30	6.5	1.30 (0.79-2.13)	0.30
rs11191979C/C × rs187304410G/A	0	0.0	2	0.4	0.19 (0.01-4.07)	0.47
		Males				
rs11191736C/C × rs34040810C/C	231	95.9	224	99.1	**0.21 (0.04-0.95)**	**0.03**
rs11191736C/C × rs34040810C/A	4	1.7	2	0.9	1.70 (0.36-8.07)	0.74
rs11191736C/T × rs34040810C/C	4	1.7	0	0.0	8.58 (0.46-160.33)	0.15
rs11191736C/T × rs34040810C/A	2	0.8	0	0.0	4.73 (0.23-99.04)	0.51
rs11191736C/C × rs2289964C/C	184	76.3	179	79.2	0.85 (0.55-1.31)	0.46
rs11191736C/C × rs2289964C/T	47	19.5	41	18.1	1.09 (0.69-1.74)	0.71
rs11191736C/C × rs2289964T/T	4	1.7	6	2.7	0.64 (0.19-2.17)	0.67
rs11191736C/T × rs2289964C/C	3	1.2	0	0.0	6.65 (0.34-129.43)	0.27
rs11191736C/T × rs2289964C/T	2	0.8	0	0.0	4.73 (0.23-99.04)	0.51
rs11191736C/T × rs2289964T/T	1	0.4	0	0.0	2.83 (0.11-69.72)	0.97
rs11191736C/C × rs11191979T/T	124	51.5	106	48.0	1.15 (0.80-1.66)	0.45
rs11191736C/C × rs11191979T/C	92	38.2	98	44.3	0.77 (0.53-1.12)	0.18
rs11191736C/C × rs11191979C/C	19	7.9	17	7.7	1.03 (0.52-2.03)	0.94
rs11191736C/T × rs11191979T/T	2	0.8	0	0.0	4.62 (0.22-96.86)	0.52
rs11191736C/T × rs11191979T/C	4	1.7	0	0.0	8.39 (0.45-156.81)	0.16
rs11191736C/C × rs187304410G/G	226	93.8	219	96.9	0.48 (0.19-1.20)	0.11
rs11191736C/C × rs187304410G/A	9	3.7	7	3.1	1.20 (0.45-3.17)	0.90
rs11191736C/T × rs187304410G/G	3	1.2	0	0.0	6.65 (0.34-129.43)	0.27
rs11191736C/T × rs187304410G/A	3	1.2	0	0.0	6.65 (0.34-129.43)	0.27
rs34040810C/C × rs2289964C/C	192	76.2	185	79.1	0.85 (0.55-1.30)	0.45
rs34040810C/C × rs2289964C/T	47	18.7	41	17.5	1.08 (0.68-1.71)	0.75
rs34040810C/C × rs2289964T/T	6	2.4	5	2.1	1.10 (0.35-3.47)	0.90
rs34040810C/A × rs2289964C/T	7	2.8	1	0.4	4.76 (0.82-27.70)	0.09
rs34040810C/A × rs2289964T/T	0	0.0	2	0.9	0.18 (0.01-3.86)	0.45
rs34040810C/C × rs11191979T/T	123	49.2	109	47.6	1.07 (0.74-1.53)	0.73
rs34040810C/C × rs11191979T/C	97	38.8	99	43.2	0.83 (0.58-1.20)	0.32
rs34040810C/C × rs11191979C/C	23	9.2	18	7.9	1.19 (0.62-2.26)	0.60
rs34040810C/A × rs11191979T/T	6	2.4	2	0.9	2.42 (0.56-10.52)	0.34
rs34040810C/A × rs11191979T/C	1	0.4	1	0.4	0.92 (0.09-8.87)	0.52
rs34040810C/C × rs187304410G/G	237	94.0	225	96.2	0.63 (0.27-1.47)	0.28
rs34040810C/C × rs187304410G/A	8	3.2	6	2.6	1.22 (0.43-3.45)	0.90
rs34040810C/A × rs187304410G/G	2	0.8	1	0.4	1.55 (0.20-11.85)	0.95
rs34040810C/A × rs187304410G/A	5	2.0	2	0.9	2.07 (0.46-9.32)	0.51
rs2289964C/C × rs11191979T/T	110	44.0	101	44.1	1.00 (0.69-1.43)	0.98
rs2289964C/C × rs11191979T/C	64	25.6	72	31.4	0.75 (0.50-1.12)	0.16
rs2289964C/C × rs11191979C/C	16	6.4	9	3.9	1.63 (0.72-3.70)	0.31
rs2289964C/T × rs11191979T/T	19	7.6	7	3.1	**2.50 (1.06-5.92)**	**0.05**
rs2289964C/T × rs11191979T/C	30	12.0	27	11.8	1.02 (0.59-1.78)	0.94
rs2289964C/T × rs11191979C/C	5	2.0	6	2.6	0.77 (0.24-2.43)	0.88
rs2289964T/T × rs11191979T/T	0	0.0	3	1.3	0.13 (0.01-2.51)	0.22
rs2289964T/T × rs11191979T/C	4	1.6	1	0.4	2.78 (0.43-17.80)	0.42
rs2289964T/T × rs11191979C/C	2	0.8	3	1.3	0.65 (0.13-3.33)	0.92
rs2289964C/C × rs187304410G/G	187	74.2	181	77.4	0.84 (0.56-1.28)	0.42
rs2289964C/C × rs187304410G/A	5	2.0	4	1.7	1.14 (0.32-4.01)	0.91
rs2289964C/T × rs187304410G/G	47	18.7	39	16.7	1.15 (0.72-1.83)	0.57
rs2289964C/T × rs187304410G/A	7	2.8	3	1.3	2.02 (0.56-7.28)	0.40

rs2289964T/T × rs187304410G/G	5	2.0	6	2.6	0.78 (0.25-2.47)	0.90
rs2289964T/T × rs187304410G/A	1	0.4	1	0.4	0.93 (0.10-8.99)	0.51
rs11191979T/T × rs187304410G/G	118	47.2	104	45.4	1.07 (0.75-1.54)	0.70
rs11191979T/T × rs187304410G/A	11	4.4	7	3.1	1.42 (0.56-3.64)	0.59
rs11191979T/C × rs187304410G/G	96	38.4	99	43.2	0.82 (0.57-1.18)	0.28
rs11191979T/C × rs187304410G/A	2	0.8	1	0.4	1.53 (0.20-11.69)	0.94
rs11191979C/C × rs187304410G/G	23	9.2	18	7.9	1.19 (0.62-2.26)	0.60
Females						
rs11191736C/C × rs34040810C/C	203	100.0	228	98.7	6.23 (0.32-121.43)	0.10
rs11191736C/T × rs34040810C/C	0	0.0	1	0.4	0.38 (0.02-9.32)	0.95
rs11191736C/T × rs34040810C/A	0	0.0	2	0.9	0.23 (0.01-4.73)	0.54
rs11191736C/C × rs2289964C/C	156	78.0	174	75.3	1.16 (0.74-1.82)	0.51
rs11191736C/C × rs2289964C/T	43	21.5	54	23.4	0.90 (0.57-1.41)	0.64
rs11191736C/C × rs2289964T/T	1	0.5	0	0.0	3.48 (0.14-85.94)	0.94
rs11191736C/T × rs2289964C/C	0	0.0	1	0.4	0.38 (0.02-9.46)	0.94
rs11191736C/T × rs2289964C/T	0	0.0	2	0.9	0.23 (0.01-4.80)	0.54
rs11191736C/C × rs11191979T/T	109	53.7	122	54.0	0.99 (0.68-1.45)	0.95
rs11191736C/C × rs11191979T/C	80	39.4	89	39.4	1.00 (0.68-1.48)	1.00
rs11191736C/C × rs11191979C/C	14	6.9	12	5.3	1.32 (0.60-2.93)	0.49
rs11191736C/T × rs11191979T/T	0	0.0	2	0.9	0.22 (0.01-4.62)	0.53
rs11191736C/T × rs11191979T/C	0	0.0	1	0.4	0.37 (0.01-9.12)	0.96
rs11191736C/C × rs187304410G/G	201	99.0	218	94.4	**5.99 (1.34-26.89)**	**0.01**
rs11191736C/C × rs187304410G/A	2	1.0	10	4.3	0.26 (0.07-1.05)	0.07
rs11191736C/T × rs187304410G/G	0	0.0	2	0.9	0.23 (0.01-4.73)	0.54
rs11191736C/T × rs187304410G/A	0	0.0	1	0.4	0.38 (0.02-9.32)	0.95
rs34040810C/C × rs2289964C/C	173	79.0	175	74.2	1.31 (0.85-2.03)	0.22
rs34040810C/C × rs2289964C/T	44	20.1	59	25.0	0.75 (0.48-1.17)	0.21
rs34040810C/C × rs2289964T/T	1	0.5	0	0.0	3.25 (0.13-80.14)	0.97
rs34040810C/A × rs2289964C/T	1	0.5	2	0.8	0.64 (0.08-4.92)	0.95
rs34040810C/C × rs11191979T/T	118	53.6	124	53.7	1.00 (0.69-1.45)	0.99
rs34040810C/C × rs11191979T/C	85	38.6	91	39.4	0.97 (0.66-1.41)	0.87
rs34040810C/C × rs11191979C/C	16	7.3	14	6.1	1.22 (0.58-2.55)	0.61
rs34040810C/A × rs11191979T/T	1	0.5	1	0.4	1.05 (0.11-10.17)	0.50
rs34040810C/A × rs11191979T/C	0	0.0	1	0.4	0.35 (0.01-8.60)	0.98
rs34040810C/C × rs187304410G/G	219	98.6	224	94.9	**3.91 (1.09-14.05)**	**0.02**
rs34040810C/C × rs187304410G/A	2	0.9	10	4.2	**0.24 (0.06-0.98)**	**0.05**
rs34040810C/A × rs187304410G/G	1	0.5	1	0.4	1.06 (0.11-10.30)	0.51
rs34040810C/A × rs187304410G/A	0	0.0	1	0.4	0.35 (0.01-8.71)	0.98
rs2289964C/C × rs11191979T/T	106	48.8	111	48.1	1.03 (0.71-1.50)	0.87
rs2289964C/C × rs11191979T/C	57	26.3	57	24.7	1.09 (0.71-1.66)	0.70
rs2289964C/C × rs11191979C/C	8	3.7	5	2.2	1.67 (0.56-4.96)	0.50
rs2289964C/T × rs11191979T/T	11	5.1	14	6.1	0.83 (0.37-1.86)	0.65
rs2289964C/T × rs11191979T/C	27	12.4	35	15.2	0.80 (0.46-1.37)	0.41
rs2289964C/T × rs11191979C/C	7	3.2	9	3.9	0.83 (0.31-2.21)	0.90
rs2289964T/T × rs11191979C/C	1	0.5	0	0.0	3.21 (0.13-79.17)	0.98
rs2289964C/C × rs187304410G/G	171	78.1	169	71.6	1.41 (0.92-2.17)	0.11
rs2289964C/C × rs187304410G/A	2	0.9	6	2.5	0.41 (0.09-1.77)	0.33
rs2289964C/T × rs187304410G/G	45	20.5	56	23.7	0.83 (0.53-1.30)	0.41
rs2289964C/T × rs187304410G/A	0	0.0	5	2.1	0.10 (0.01-1.74)	0.09
rs2289964T/T × rs187304410G/G	1	0.5	0	0.0	3.25 (0.13-80.14)	0.97
rs11191979T/T × rs187304410G/G	118	53.6	119	51.5	1.09 (0.75-1.58)	0.65
rs11191979T/T × rs187304410G/A	1	0.5	6	2.6	0.24 (0.04-1.41)	0.14
rs11191979T/C × rs187304410G/G	84	38.2	89	38.5	0.99 (0.67-1.44)	0.94

rs11191979T/C × rs187304410G/A	1	0.5	3	1.3	0.45 (0.07-3.05)	0.65
rs11191979C/C × rs187304410G/G	16	7.3	12	5.2	1.43 (0.66-3.10)	0.36
rs11191979C/C × rs187304410G/A	0	0.0	2	0.9	0.21 (0.01-4.36)	0.50

[1] Absolute number of individuals with particular genotype combination (minor alleles in genotypes are underlined).

[2] Percentage of individuals with particular genotype combination.

[3] OR, odds ratio; CI, confidence interval. Bold are statistically significant P-values.

Supplementary Table S3 *GSTO1* by *GCLC* genotype combinations and their associations with psoriasis risk in the entire groups

GSTO1 × *GCLC* genotype combinations	Patients		Controls		OR (95% CI)[3]	*P*-value
	n[1]	%[2]	n[1]	%[2]		
rs11191736C/C × rs524553C/C	263	59.6	264	58.0	1.07 (0.82-1.39)	0.62
rs11191736C/C × rs524553C/T	157	35.6	166	36.5	0.96 (0.73-1.26)	0.78
rs11191736C/C × rs524553T/T	15	3.4	22	4.8	0.69 (0.35-1.35)	0.28
rs11191736C/T × rs524553C/C	2	0.5	1	0.2	1.72 (0.23-13.10)	0.98
rs11191736C/T × rs524553C/T	4	0.9	2	0.4	1.87 (0.40-8.81)	0.65
rs11191736C/C × rs542914C/C	158	35.7	164	36.0	0.99 (0.75-1.30)	0.95
rs11191736C/C × rs542914C/A	223	50.5	216	47.4	1.13 (0.87-1.47)	0.36
rs11191736C/C × rs542914A/A	55	12.4	73	16.0	0.75 (0.51-1.09)	0.13
rs11191736C/T × rs542914C/C	2	0.5	1	0.2	1.72 (0.23-13.10)	0.98
rs11191736C/T × rs542914C/A	4	0.9	2	0.4	1.87 (0.40-8.80)	0.65
rs11191736C/C × rs648595G/G	70	15.8	94	20.7	0.72 (0.51-1.01)	0.06
rs11191736C/C × rs648595G/T	234	52.8	216	47.5	1.24 (0.95-1.61)	0.11
rs11191736C/C × rs648595T/T	133	30.0	142	31.2	0.95 (0.71-1.26)	0.70
rs11191736C/T × rs648595G/G	0	0.0	1	0.2	0.34 (0.01-8.41)	0.99
rs11191736C/T × rs648595G/T	4	0.9	1	0.2	3.10 (0.49-19.78)	0.35
rs11191736C/T × rs648595T/T	2	0.5	1	0.2	1.72 (0.23-13.04)	0.98
rs11191736C/C × rs6933870C/C	150	33.9	155	33.9	1.00 (0.76-1.32)	0.99
rs11191736C/C × rs6933870C/G	222	50.2	228	49.9	1.01 (0.78-1.32)	0.92
rs11191736C/C × rs6933870G/G	64	14.5	71	15.5	0.92 (0.64-1.33)	0.66
rs11191736C/T × rs6933870C/C	2	0.5	1	0.2	1.73 (0.23-13.13)	0.98
rs11191736C/T × rs6933870C/G	4	0.9	1	0.2	3.12 (0.49-19.91)	0.35
rs11191736C/T × rs6933870G/G	0	0.0	1	0.2	0.34 (0.01-8.46)	0.99
rs11191736C/C × rs2397147T/T	178	40.2	177	39.0	1.05 (0.80-1.37)	0.71
rs11191736C/C × rs2397147T/C	216	48.8	221	48.7	1.00 (0.77-1.30)	0.98
rs11191736C/C × rs2397147C/C	43	9.7	53	11.7	0.81 (0.53-1.24)	0.34
rs11191736C/T × rs2397147T/T	2	0.5	1	0.2	1.71 (0.23-13.02)	0.98
rs11191736C/T × rs2397147T/C	4	0.9	2	0.4	1.85 (0.39-8.75)	0.66
rs11191736C/C × rs17883901G/G	370	87.9	326	88.3	0.96 (0.62-1.47)	0.84
rs11191736C/C × rs17883901G/A	44	10.5	35	9.5	1.11 (0.70-1.78)	0.65
rs11191736C/C × rs17883901A/A	1	0.2	6	1.6	0.20 (0.03-1.18)	0.09
rs11191736C/T × rs17883901G/G	6	1.4	2	0.5	2.30 (0.53-9.96)	0.38
rs34040810C/C × rs524553C/C	281	59.7	270	57.7	1.08 (0.84-1.41)	0.54
rs34040810C/C × rs524553C/T	167	35.5	171	36.5	0.95 (0.73-1.25)	0.73
rs34040810C/C × rs524553T/T	15	3.2	22	4.7	0.67 (0.34-1.30)	0.23
rs34040810C/A × rs524553C/C	4	0.8	3	0.6	1.28 (0.31-5.21)	0.99
rs34040810C/A × rs524553C/T	4	0.8	2	0.4	1.80 (0.38-8.47)	0.69
rs34040810C/C × rs542914C/C	172	36.4	166	35.4	1.05 (0.80-1.37)	0.74
rs34040810C/C × rs542914C/A	234	49.6	224	47.8	1.08 (0.83-1.39)	0.58
rs34040810C/C × rs542914A/A	58	12.3	74	15.8	0.75 (0.52-1.08)	0.12
rs34040810C/A × rs542914C/C	2	0.4	2	0.4	0.99 (0.17-5.76)	0.62
rs34040810C/A × rs542914C/A	6	1.3	3	0.6	1.86 (0.50-6.85)	0.51
rs34040810C/C × rs648595G/G	72	15.2	95	20.3	**0.70 (0.50-0.99)**	**0.04**
rs34040810C/C × rs648595G/T	250	52.9	223	47.6	1.23 (0.95-1.59)	0.11
rs34040810C/C × rs648595T/T	143	30.2	145	31.0	0.97 (0.73-1.27)	0.80
rs34040810C/A × rs648595G/G	5	1.1	1	0.2	3.66 (0.60-22.36)	0.22
rs34040810C/A × rs648595G/T	2	0.4	2	0.4	0.99 (0.17-5.74)	0.62
rs34040810C/A × rs648595T/T	1	0.2	2	0.4	0.59 (0.08-4.50)	0.99
rs34040810C/C × rs6933870C/C	162	34.3	158	33.6	1.03 (0.79-1.35)	0.82
rs34040810C/C × rs6933870C/G	235	49.8	235	50.0	0.99 (0.77-1.28)	0.95

rs34040810C/C × rs6933870G/G	67	14.2	72	15.3	0.91 (0.64-1.31)	0.63
rs34040810C/A × rs6933870C/C	1	0.2	2	0.4	0.60 (0.08-4.53)	1.00
rs34040810C/A × rs6933870C/G	5	1.1	2	0.4	2.20 (0.49-9.89)	0.45
rs34040810C/A × rs6933870C/C	2	0.4	1	0.2	1.66 (0.22-12.64)	1.00
rs34040810C/C × rs2397147T/T	196	41.4	181	38.8	1.12 (0.86-1.45)	0.40
rs34040810C/C × rs2397147T/C	224	47.4	228	48.8	0.94 (0.73-1.22)	0.65
rs34040810C/C × rs2397147C/C	45	9.5	53	11.3	0.82 (0.54-1.25)	0.36
rs34040810C/A × rs2397147T/T	2	0.4	2	0.4	0.99 (0.17-5.73)	0.63
rs34040810C/A × rs2397147T/C	6	1.3	3	0.6	1.85 (0.50-6.81)	0.52
rs34040810C/C × rs17883901G/G	382	87.8	331	88.3	0.96 (0.63-1.47)	0.84
rs34040810C/C × rs17883901G/A	42	9.7	35	9.3	1.04 (0.65-1.66)	0.88
rs34040810C/C × rs17883901A/A	3	0.7	6	1.6	0.46 (0.12-1.70)	0.37
rs34040810C/A × rs17883901G/G	6	1.4	3	0.8	1.61 (0.44-5.95)	0.65
rs34040810C/A × rs17883901G/A	2	0.5	0	0.0	4.33 (0.21-90.50)	0.55
rs2289964C/C × rs524553C/C	224	47.9	201	42.9	1.22 (0.94-1.58)	0.13
rs2289964C/C × rs524553C/T	128	27.4	139	29.7	0.89 (0.67-1.18)	0.43
rs2289964C/C × rs524553T/T	11	2.4	18	3.8	0.60 (0.28-1.29)	0.19
rs2289964C/T × rs524553C/C	56	12.0	65	13.9	0.84 (0.57-1.24)	0.38
rs2289964C/T × rs524553C/T	39	8.3	34	7.3	1.16 (0.72-1.87)	0.54
rs2289964C/T × rs524553T/T	3	0.6	4	0.9	0.78 (0.19-3.16)	1.00
rs2289964T/T × rs524553C/C	5	1.1	7	1.5	0.73 (0.24-2.21)	0.77
rs2289964T/T × rs524553C/T	2	0.4	0	0.0	5.02 (0.24-104.9)	0.48
rs2289964C/C × rs542914C/C	134	28.5	127	27.1	1.07 (0.81-1.43)	0.62
rs2289964C/C × rs542914C/A	182	38.7	175	37.3	1.06 (0.82-1.38)	0.66
rs2289964C/C × rs542914A/A	48	10.2	57	12.2	0.82 (0.55-1.24)	0.35
rs2289964C/T × rs542914C/C	37	7.9	38	8.1	0.97 (0.60-1.55)	0.90
rs2289964C/T × rs542914C/A	53	11.3	48	10.2	1.11 (0.74-1.68)	0.61
rs2289964C/T × rs542914A/A	9	1.9	17	3.6	0.53 (0.24-1.19)	0.16
rs2289964T/T × rs542914C/C	2	0.4	3	0.6	0.71 (0.14-3.62)	1.00
rs2289964T/T × rs542914C/A	4	0.9	4	0.9	1.00 (0.27-3.71)	0.72
rs2289964T/T × rs542914A/A	1	0.2	0	0.0	3.00 (0.12-73.84)	1.00
rs2289964C/C × rs648595G/G	59	12.6	70	15.0	0.82 (0.56-1.18)	0.29
rs2289964C/C × rs648595G/T	192	40.9	181	38.7	1.10 (0.84-1.42)	0.50
rs2289964C/C × rs648595T/T	113	24.0	108	23.1	1.06 (0.78-1.43)	0.73
rs2289964C/T × rs648595G/G	17	3.6	26	5.6	0.64 (0.34-1.19)	0.16
rs2289964C/T × rs648595G/T	53	11.3	40	8.5	1.36 (0.88-2.09)	0.16
rs2289964C/T × rs648595T/T	29	6.2	36	7.7	0.79 (0.48-1.31)	0.36
rs2289964T/T × rs648595G/T	5	1.1	4	0.9	1.22 (0.35-4.27)	0.99
rs2289964T/T × rs648595T/T	2	0.4	3	0.6	0.71 (0.14-3.62)	1.00
rs2289964C/C × rs6933870C/C	124	26.4	120	25.5	1.05 (0.78-1.40)	0.77
rs2289964C/C × rs6933870C/G	183	38.9	185	39.4	0.98 (0.76-1.28)	0.89
rs2289964C/C × rs6933870G/G	57	12.1	55	11.7	1.04 (0.70-1.55)	0.84
rs2289964C/T × rs6933870C/C	36	7.7	37	7.9	0.97 (0.60-1.57)	0.90
rs2289964C/T × rs6933870C/G	53	11.3	48	10.2	1.12 (0.74-1.69)	0.60
rs2289964C/T × rs6933870G/G	10	2.1	18	3.8	0.55 (0.25-1.20)	0.12
rs2289964T/T × rs6933870C/C	3	0.6	3	0.6	1.00 (0.23-4.42)	0.68
rs2289964T/T × rs6933870C/G	4	0.9	4	0.9	1.00 (0.27-3.72)	0.72
rs2289964C/C × rs2397147T/T	150	31.9	134	28.7	1.16 (0.88-1.54)	0.28
rs2289964C/C × rs2397147T/C	178	37.9	183	39.2	0.95 (0.73-1.23)	0.68
rs2289964C/C × rs2397147C/C	36	7.7	40	8.6	0.89 (0.55-1.42)	0.61
rs2289964C/T × rs2397147T/T	44	9.4	46	9.9	0.95 (0.61-1.46)	0.80
rs2289964C/T × rs2397147T/C	48	10.2	44	9.4	1.09 (0.71-1.68)	0.68
rs2289964C/T × rs2397147C/C	7	1.5	13	2.8	0.54 (0.22-1.34)	0.25

rs2289964T/T × rs2397147T/T	3	0.6	3	0.6	0.99 (0.22-4.40)	0.69
rs2289964T/T × rs2397147T/C	4	0.9	4	0.9	0.99 (0.27-3.69)	0.73
rs2289964C/C × rs17883901G/G	296	68.5	259	69.1	0.97 (0.72-1.31)	0.87
rs2289964C/C × rs17883901G/A	35	8.1	22	5.9	1.41 (0.81-2.46)	0.22
rs2289964C/C × rs17883901A/A	2	0.5	4	1.1	0.48 (0.10-2.26)	0.56
rs2289964C/T × rs17883901G/G	83	19.2	69	18.4	1.05 (0.74-1.50)	0.77
rs2289964C/T × rs17883901G/A	8	1.9	13	3.5	0.54 (0.23-1.28)	0.22
rs2289964C/T × rs17883901A/A	1	0.2	2	0.5	0.52 (0.07-3.95)	0.90
rs2289964T/T × rs17883901G/G	6	1.4	6	1.6	0.87 (0.29-2.59)	0.96
rs2289964T/T × rs17883901G/A	1	0.2	0	0.0	2.61 (0.11-64.28)	0.94
rs11191979T/T × rs524553C/C	144	30.8	122	26.6	1.23 (0.92-1.63)	0.16
rs11191979T/T × rs524553C/T	94	20.1	102	22.3	0.88 (0.64-1.21)	0.43
rs11191979T/T × rs524553T/T	10	2.1	10	2.2	0.98 (0.40-2.38)	0.96
rs11191979T/C × rs524553C/C	113	24.2	122	26.6	0.88 (0.65-1.18)	0.39
rs11191979T/C × rs524553C/T	63	13.5	59	12.9	1.05 (0.72-1.54)	0.78
rs11191979T/C × rs524553T/T	5	1.1	11	2.4	0.46 (0.17-1.29)	0.19
rs11191979C/C × rs524553C/C	26	5.6	22	4.8	1.17 (0.65-2.09)	0.60
rs11191979C/C × rs524553C/T	12	2.6	9	2.0	1.30 (0.55-3.05)	0.69
rs11191979C/C × rs524553T/T	0	0.0	1	0.2	0.33 (0.01-8.03)	0.99
rs11191979T/T × rs542914C/C	95	20.3	69	15.0	**1.44 (1.02-2.02)**	**0.04**
rs11191979T/T × rs542914C/A	121	25.9	126	27.5	0.92 (0.69-1.23)	0.58
rs11191979T/T × rs542914A/A	30	6.4	40	8.7	0.72 (0.44-1.17)	0.18
rs11191979T/C × rs542914C/C	60	12.8	81	17.6	**0.69 (0.48-0.99)**	**0.04**
rs11191979T/C × rs542914C/A	99	21.2	83	18.1	1.22 (0.88-1.68)	0.24
rs11191979T/C × rs542914A/A	24	5.1	28	6.1	0.83 (0.47-1.46)	0.52
rs11191979C/C × rs542914C/C	17	3.6	14	3.1	1.20 (0.58-2.46)	0.62
rs11191979C/C × rs542914C/A	18	3.8	14	3.1	1.27 (0.62-2.59)	0.51
rs11191979C/C × rs542914A/A	4	0.9	4	0.9	0.98 (0.26-3.65)	0.74
rs11191979T/T × rs648595G/G	50	10.7	47	10.3	1.04 (0.69-1.59)	0.84
rs11191979T/T × rs648595G/T	125	26.7	127	27.7	0.95 (0.71-1.26)	0.71
rs11191979T/T × rs648595T/T	72	15.4	62	13.5	1.16 (0.80-1.67)	0.43
rs11191979T/C × rs648595G/G	23	4.9	39	8.5	**0.55 (0.33-0.94)**	**0.03**
rs11191979T/C × rs648595G/T	102	21.7	82	17.9	1.27 (0.92-1.76)	0.14
rs11191979T/C × rs648595T/T	58	12.4	70	15.3	0.78 (0.54-1.14)	0.20
rs11191979C/C × rs648595G/G	3	0.6	6	1.3	0.52 (0.14-1.93)	0.48
rs11191979C/C × rs648595G/T	22	4.7	12	2.6	1.83 (0.89-3.74)	0.09
rs11191979C/C × rs648595T/T	14	3.0	13	2.8	1.05 (0.49-2.27)	0.89
rs11191979T/T × rs6933870C/C	77	16.5	70	15.2	1.10 (0.77-1.56)	0.61
rs11191979T/T × rs6933870C/G	128	27.4	128	27.8	0.98 (0.73-1.30)	0.87
rs11191979T/T × rs6933870G/G	43	9.2	38	8.3	1.12 (0.71-1.77)	0.62
rs11191979T/C × rs6933870C/C	69	14.7	71	15.4	0.95 (0.66-1.36)	0.77
rs11191979T/C × rs6933870C/G	90	19.2	94	20.4	0.93 (0.67-1.28)	0.65
rs11191979T/C × rs6933870G/G	22	4.7	27	5.9	0.79 (0.44-1.41)	0.43
rs11191979C/C × rs6933870C/C	17	3.6	15	3.3	1.12 (0.55-2.27)	0.76
rs11191979C/C × rs6933870C/G	19	4.1	12	2.6	1.58 (0.76-3.29)	0.22
rs11191979C/C × rs6933870G/G	3	0.6	5	1.1	0.62 (0.16-2.39)	0.70
rs11191979T/T × rs2397147T/T	99	21.1	77	16.8	1.32 (0.95-1.84)	0.10
rs11191979T/T × rs2397147T/C	122	26.0	130	28.4	0.88 (0.66-1.18)	0.41
rs11191979T/T × rs2397147C/C	26	5.5	27	5.9	0.93 (0.54-1.63)	0.81
rs11191979T/C × rs2397147T/T	79	16.8	85	18.6	0.89 (0.63-1.24)	0.48
rs11191979T/C × rs2397147T/C	88	18.8	85	18.6	1.01 (0.73-1.41)	0.95
rs11191979T/C × rs2397147C/C	16	3.4	21	4.6	0.73 (0.38-1.42)	0.36
rs11191979C/C × rs2397147T/T	18	3.8	17	3.7	1.03 (0.53-2.03)	0.92

rs11191979C/C × rs2397147T/C	18	3.8	12	2.6	1.48 (0.70-3.11)	0.30
rs11191979C/C × rs2397147C/C	3	0.6	3	0.7	0.97 (0.22-4.31)	0.71
rs11191979T/T × rs17883901G/G	199	45.7	171	46.2	0.98 (0.74-1.30)	0.89
rs11191979T/T × rs17883901G/A	30	6.9	15	4.1	1.75 (0.93-3.31)	0.08
rs11191979T/T × rs17883901A/A	1	0.2	3	0.8	0.36 (0.05-2.47)	0.51
rs11191979T/C × rs17883901G/G	160	36.8	135	36.5	1.01 (0.76-1.35)	0.93
rs11191979T/C × rs17883901G/A	11	2.5	18	4.9	0.51 (0.24-1.09)	0.08
rs11191979T/C × rs17883901A/A	0	0.0	2	0.5	0.17 (0.01-3.54)	0.41
rs11191979C/C × rs17883901G/G	29	6.7	25	6.8	0.99 (0.57-1.72)	0.96
rs11191979C/C × rs17883901G/A	3	0.7	1	0.3	1.99 (0.29-13.57)	0.73
rs11191979C/C × rs17883901A/A	2	0.5	0	0.0	4.27 (0.20-89.30)	0.55
rs187304410G/G × rs524553C/C	277	58.8	264	56.4	1.10 (0.85-1.43)	0.46
rs187304410G/G × rs524553C/T	165	35.0	165	35.3	0.99 (0.76-1.29)	0.94
rs187304410G/G × rs524553T/T	14	3.0	20	4.3	0.69 (0.34-1.38)	0.29
rs187304410G/A × rs524553C/C	8	1.7	9	1.9	0.89 (0.35-2.26)	0.99
rs187304410G/A × rs524553C/T	6	1.3	8	1.7	0.76 (0.27-2.12)	0.78
rs187304410G/A × rs524553C/C	1	0.2	2	0.4	0.59 (0.08-4.52)	1.00
rs187304410G/G × rs542914C/C	168	35.6	162	34.5	1.05 (0.80-1.37)	0.74
rs187304410G/G × rs542914C/A	232	49.2	218	46.5	1.11 (0.86-1.44)	0.41
rs187304410G/G × rs542914A/A	57	12.1	70	14.9	0.78 (0.54-1.14)	0.20
rs187304410G/A × rs542914C/C	6	1.3	6	1.3	0.99 (0.33-2.97)	0.78
rs187304410G/A × rs542914C/A	8	1.7	9	1.9	0.89 (0.35-2.26)	0.99
rs187304410G/A × rs542914A/A	1	0.2	4	0.9	0.33 (0.05-2.10)	0.37
rs187304410G/G × rs648595G/G	72	15.2	90	19.2	0.75 (0.54-1.06)	0.10
rs187304410G/G × rs648595G/T	247	52.2	219	46.8	1.24 (0.96-1.61)	0.10
rs187304410G/G × rs648595T/T	139	29.4	141	30.1	0.97 (0.73-1.28)	0.80
rs187304410G/A × rs648595G/G	5	1.1	6	1.3	0.84 (0.27-2.62)	0.99
rs187304410G/A × rs648595G/T	5	1.1	6	1.3	0.84 (0.27-2.62)	0.99
rs187304410G/A × rs648595T/T	5	1.1	6	1.3	0.84 (0.27-2.62	0.99
rs187304410G/G × rs6933870C/C	158	33.5	154	32.8	1.03 (0.79-1.35)	0.82
rs187304410G/G × rs6933870C/G	231	48.9	230	48.9	1.00 (0.77-1.29)	1.00
rs187304410G/G × rs6933870G/G	68	14.4	67	14.3	1.01 (0.70-1.46)	0.95
rs187304410G/A × rs6933870C/C	5	1.1	6	1.3	0.84 (0.27-2.64)	0.99
rs187304410G/A × rs6933870C/G	9	1.9	7	1.5	1.27 (0.48-3.33)	0.81
rs187304410G/A × rs6933870G/G	1	0.2	6	1.3	0.23 (0.04-1.35)	0.13
rs187304410G/G × rs2397147T/T	193	40.8	176	37.7	1.14 (0.88-1.48)	0.33
rs187304410G/G × rs2397147T/C	220	46.5	223	47.8	0.95 (0.74-1.23)	0.70
rs187304410G/G × rs2397147C/C	45	9.5	49	10.5	0.90 (0.59-1.37)	0.62
rs187304410G/A × rs2397147T/T	5	1.1	7	1.5	0.72 (0.24-2.18)	0.75
rs187304410G/A × rs2397147T/C	10	2.1	8	1.7	1.22 (0.49-3.05)	0.83
rs187304410G/A × rs2397147C/C	0	0.0	4	0.9	0.11 (0.01-2.03)	0.13
rs187304410G/G × rs17883901G/G	378	86.9	321	85.6	1.12 (0.75-1.67)	0.59
rs187304410G/G × rs17883901G/A	40	9.2	34	9.1	1.02 (0.63-1.64)	0.95
rs187304410G/G × rs17883901A/A	3	0.7	5	1.3	0.55 (0.14-2.10)	0.57
rs187304410G/A × rs17883901G/G	10	2.3	13	3.5	0.66 (0.28-1.51)	0.32
rs187304410G/A × rs17883901G/A	4	0.9	1	0.3	2.60 (0.41-16.61)	0.46
rs187304410G/A × rs17883901A/A	0	0.0	1	0.3	0.29 (0.01-7.06)	0.94

[1] Absolute number of individuals with particular genotype combination (minor alleles in genotypes are underlined).

[2] Percentage of individuals with particular genotype combination.

[3] OR, odds ratio; CI, confidence interval. Bold are statistically significant P-values.

Supplementary Table S4 The best *n*-order mbmdr-models of SNP × risk factor interactions significantly associated with psoriasis risk in the entire groups

	Mbmdr-models of SNP × risk factors interactions	NH	β-H	WH	NL	β-L	WL	P_{perm}
	Two-order models							
1	ALCOHOL × *GSTO1* rs34040810	1	0 .302	41 .60	1	-0 .303	44 .57	**<0.0001**
2	ALCOHOL × *GCLC* rs542914	3	0 .304	42 .30	2	-0 .152	18 .77	**<0.0001**
3	ALCOHOL × SMOKE	2	0 .303	42 .27	1	-0 .130	13 .15	**<0.0001**
4	ALCOHOL × *GCLC* rs648595	3	0 .303	42 .16	2	-0 .158	19 .69	**<0.0001**
5	ALCOHOL × *GCLC* rs2397147	3	0 .302	41 .90	2	-0 .150	18 .49	**<0.0001**
	Three-order models							
1	ALCOHOL × SMOKE × *GCLC* rs17883901	2	0 .277	30 .57	2	-0 .172	17 .51	**<0.0001**
2	ALCOHOL × SMOKE × *GCLC* rs2397147	5	0 .298	39 .91	1	-0 .105	7 .29	**<0.0001**
3	ALCOHOL × SMOKE × *GCLC* rs6933870	5	0 .310	38 .88	2	-0 .118	10 .80	**<0.0001**
4	ALCOHOL × SMOKE × *GCLC* rs648595	5	0 .318	40 .45	3	-0 .152	18 .78	**<0.0001**
5	ALCOHOL × SMOKE × *GCLC* rs542914	4	0 .305	36 .71	2	-0 .180	17 .62	**<0.0001**
	Four-order models							
1	ALCOHOL × SMOKE × *GSTO1* rs34040810 × *GSTO1* rs11191736	2	0 .303	38 .40	2	-0 .306	42 .89	**<0.0001**
2	ALCOHOL × *GSTO1* rs187304410 × *GSTO1* rs34040810 × *GSTO1* rs11191736	1	0 .299	36 .39	2	-0 .306	42 .89	**<0.0001**
3	ALCOHOL × SMOKE × *GCLC* rs648595 × *GSTO1* rs34040810	5	0 .317	39 .79	3	-0 .155	19 .69	**<0.0001**
4	ALCOHOL × *GCLC* rs542914 × *GSTO1* rs187304410 × *GSTO1* rs34040810	3	0 .299	39 .63	4	-0 .159	20 .56	**<0.0001**
5	ALCOHOL × SMOKE × *GSTO1* rs187304410 × *GSTO1* rs34040810	2	0 .299	39 .61	2	-0 .133	13 .80	**<0.0001**

NH—number of interacting high-risk variables (SNP and risk factors); *β*-H—regression coefficient for high-risk interactions identified in step 2 of the analysis. WH—Wald statistics for high-risk interactions; NL is the number of interacting low-risk variables; *β*-L—regression coefficient for low-risk interactions identified in step 2 of the analysis; WL—Wald statistics for low-risk interactions. P_{perm}—permutation significance levels for the models associated with psoriasis risk.
